# The reliability of assistance systems modulates the sense of control and acceptability of human operators

**DOI:** 10.1038/s41598-023-41253-8

**Published:** 2023-09-02

**Authors:** Quentin Vantrepotte, Valérian Chambon, Bruno Berberian

**Affiliations:** 1 Institut Jean Nicod, Département d’Études Cognitives, École Normale Supérieure, CNRS, PSL University, Paris, France; 2 Information Processing and Systems, ONERA, Base Aérienne 701, Salon Cedex, Salon de Provence France

**Keywords:** Psychology and behaviour, Aerospace engineering

## Abstract

Individuals are increasingly required to interact with complex and autonomous technologies, which often has a significant impact on the control they experience over their actions and choices. A better characterization of the factors responsible for modulating the control experience of human operators is therefore a major challenge to improve the quality of human-system interactions. Using a decision-making task performed in interaction with an automated system, we investigated the influence of two key properties of automated systems, their reliability and explicability, on participants' sense of agency (SoA), as well as the perceived acceptability of system choices. The results show an increase in SoA associated with the most explicable system. Importantly, the increase in system explicability influenced participants' ability to regulate the control resources they engaged in the current decision. In particular, we observed that participants' SoA varied with system reliability in the "explained" condition, whereas no variation was observed in the "non-explained" condition. Finally, we found that system reliability had a direct impact on system acceptability, such that the most reliable systems were also considered the most acceptable systems. These results highlight the importance of studying agency in human–computer interaction in order to define more acceptable automation technologies.

## Introduction

Healthy adults generally feel in control of their own actions and the effects of those actions—a feeling that is sometimes also referred to as “sense of agency”^1^. The importance of the sense of agency in human life cannot be understated. The ability to envision oneself as an agent, endowed with the capacity to bring about changes in the external environment, plays a key role in assigning moral and legal responsibility^2^ and would serve as a key motivational force for human behaviour^3–5^.

The potential impact of technology on human operators' sense of control has received increasing attention in recent years. Indeed, individuals are increasingly required to interact with more or less complex and autonomous technologies. A paradigmatic example of such human–machine interaction is embodied by the relationship between an airplane pilot and the flight assistance system. In the cockpit, the increasing level of automation introduced by the assistance system can sometimes generate ambiguities as to who controls the aircraft—the pilot themselves or the automated flight assistance system. In a pioneering study, Berberian and colleagues^6^ experimentally demonstrated this detrimental influence of automation on sense of agency (SoA). Using a flight simulator, they investigated the evolution of participants’ SoA during an aircraft navigation task with different levels of automation. They demonstrated a decrease in SoA as the level of automation increased, both at the explicit (verbal report) and implicit levels (temporal binding measure)^6^.

The opposite, however, has also been observed. According to Wen et al.^7^, being assisted during a pointing task can potentially *enhance* agentive experience. The authors used a pointing task in which participants were assisted by a computer, but only relevant commands from the participant were taken into account, with incorrect commands being ignored, so that the participant did not have full control over pointing movements. The results showed that computer assistance significantly increased participants' SoA compared to the condition in which all participant commands were executed (see also^8,9^). This apparent contradiction reflects the paradoxical effects of automation on human behavior: automation can induce an effective loss of control in the human operator, while improving performance^10^. Ueda and colleagues^9^ suggest that the effect of automation on SoA is in fact non-linear: while the assistance provided by automation increases operator performance, and therefore SoA, for low to medium levels of automation, the effect becomes detrimental to operators' SoA when the level of automation exceeds 90%, even though their performance continues to increase—the reason being that at these levels of automation, participants are no longer able to attribute the (good) performance of the system to themselves.

While these results underline the flexible nature of SoA, they also demonstrate that automation often has a significant impact on the experience of the operators in charge of controlling them. This complex relationship between automation and agentive experience can also have dramatic consequences in terms of system acceptability, operator engagement and performance^10^. In this paper, we assume that a better characterization of the relationship between automation and sense of agency could help identify guidelines on how to enhance operators' SoA in interacting with technological systems. For example, a recent line of research proposes to mitigate the decrease in SoA induced by automation by reducing system opacity. System opacity refers to the difficulty for human operators to understand how intelligent systems work, which can make their objectives confusing or difficult to represent, and their future behavior difficult to predict. Critically, system opacity has been proposed as a potential mediator of these changes in SoA when interacting with automation technology^6^. Remedying this opacity by increasing the predictability of the system's actions (i.e., the human operator's ability to anticipate the system's actions or choices) could therefore improve the SoA of human operators in charge of their control. For example, Le Goff and colleagues^12^ used the communication of system action intentions to increase system predictability, and showed that such communication improved operator SoA and performance, as well as system acceptability. Moreover, this type of communication would also have an impact on trust in artificial agents, as well as on willingness to cooperate with these agents^12^.

In a more recent study, we found a similar effect using metacognitive information feedback during decision making^13^. For an artificial system, metacognitive information refers to what the system understands about its own internal procedures—and more generally refers to the system's ability to monitor its operation and report on the product of this monitoring, in the form of a measure such as confidence in its response^14,15^. We designed an avoidance task in which an assistance system could impose a choice and communicate its confidence in that choice. Participants' choice as well as participant's SoA (implicit and explicit) and acceptability in the system’s decision were collected throughout the experiment. The results showed that the metacognitive information provided by the system helped restore the operator's SoA, and this increased SoA was also associated with greater system acceptability. In contrast, and as expected, our measure of “control used” (i.e. the resources the subject reported having invested in the task) showed a negative relationship with the explicability factor. These results complement those obtained previously^11^ and demonstrate the positive role of system intelligibility on the SoA of operators interacting with automated systems.

Interestingly, we also observed an unexpected effect of task difficulty on SoA. In particular, we found an increase in SoA with increasing difficulty, a relationship similar to that observed between difficulty and our measure of control used. This finding suggests that cognitive resources—as measured by reports of "control used"—can modify individuals’ SoA. This relationship between cognitive resources and control experience is not new, but conflicting results have been reported in this regard so far^13,16,17^. Further, we also found that task difficulty modulates the relationship between explicability and automation. When the subject acts alone and receives no assistance, the uncertainty related to the difficulty of the task prevents the formation of a reliable SoA. On the other hand, when the subject is assisted and the assistance system is well calibrated, both in terms of objective performance and metacognitive evaluation, the subject's SoA naturally benefits from the cues provided by the system. Together, these results suggest that (1) the SoA can be influenced by the level of cognitive control invested in the task, and that (2) metacognitive information (e.g. confidence) communicated by a support system can help the human operator control the amount of cognitive resources to invest in the task. Note that a recent study^18^ similarly suggests that the decision to invest cognitive resources in a task is mediated at a higher-order level through metacognitive control.

Taken together, our results support the idea that the explicability of the system (i.e., the metacognitive information it returns) guides the allocation of cognitive resources needed to maintain control over the action, and that this level of cognitive control impacts the participant’s SoA. If this is the case, the control resources invested in the task and, through them, the participant’s SoA, should be modulated by the system’ reliability (i.e., the probability that the system will provide accurate information). Furthermore, this relation between reliability and control resources should be modulated by the explicability of the system. The present study sought to test these hypotheses directly, by selectively manipulating the reliability of an assistance system and measuring the impact of this manipulation on human participants’ SoA and acceptability.

## Methods

The experimental task described in this work is based in part on a previous study^13^. In what follows, we focus on presenting the essential information needed to understand the current study, and refer to the original article for a full description of the methods.

### Participants

Forty participants were recruited (27 females, mean age = 32.4; SD = 10.4). Sample size calculation was based on the effects observed in a previous study using a similar protocol^13^. A priori power calculation was performed using G*Power software^19^, with a power of 0.80 and two-sided alpha level set at 0.05. The number of participants required to detect a mean effect size of d = 0.4 (based on a similar study^13^) in a pairwise comparison, with exclusion of ~ 10% of the sample on the basis of predefined exclusion criteria, was forty. In practice, 40 participants were recruited to reach 34 analyzable subjects (6 excluded) on the basis of our exclusion criteria (see “Data analysis” section for more details). The study was approved by the Inserm Ethics Evaluation Committee (CEEI, n. 21-810). All participants gave written informed consent before inclusion in the study, which was carried out in accordance with the declaration of Helsinki^20,21^. The inclusion criteria were being older than 18 years, reporting no history of neurological or psychiatric disorders, no auditory disorders, and a normal or corrected-to-normal vision. Participants were all naive to the purpose of the study.

The studies were conducted on the experimental platform (PRISME) of the Institut du Cerveau et de la Moelle épinière (ICM) in Paris. The participants were tested in groups (maximum 12 participants) in a large testing room designed for this purpose. Each participant was brought individually in front of a computer, isolated by partitions, and was equipped with noise-cancelling headphones. The study was divided into two sessions, on two different days to reduce the effect of fatigue. Each session lasted 2 h, and participants were paid 40 euros after completing both sessions. The procedure was the same for both sessions 1 and 2 (training and calibration phases, followed by the experimental task).

### Material and stimuli

The participants were seated in front of a screen at approximatively 60 cm (HP 23i; resolution: 1980 × 1080, 60 Hz). A keyboard and a noise cancelling headphone were used to perform the experiment. As in the previous study, the experiment consisted of a combination of an avoidance task, derived from the Random Dot Kinematogram (RDK) experimental protocol, and a temporal estimation task between an action and its effect, used as an implicit measure of participants' sense of control^22^. The stimuli presented to participants were identical in every respect to those used previously (for details, see^13^). Matlab R2016b (MathWorks Inc.) and the Psychophysics Toolbox^23–25^ were used to develop and run the experiment.

### General procedure

#### Training and calibration phases

Each session was split in two experimental blocks. The first block consisted of two training sessions and one calibration phase. The training sessions (15 trials each) were designed to familiarize the participant with the avoidance task and the temporal estimation task, whereas the calibration phase was used to estimate a psychometric curve for each participant based on RDKs.

**Figure 1:**
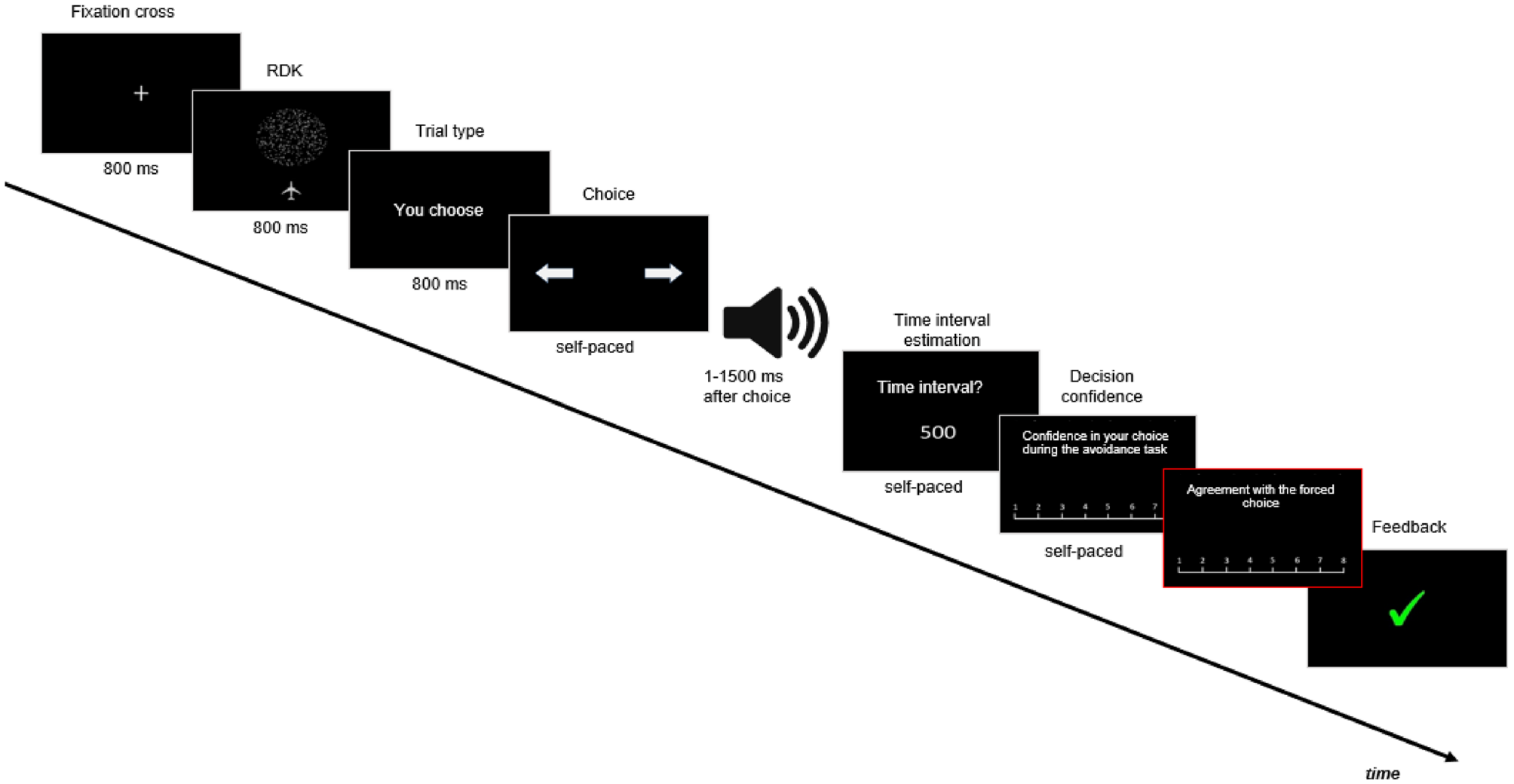
Illustration of a typical trial during the experimental phase. A fixation cross indicated the start of the trial during 800 ms. Then, a RDK appeared for a short period (800 ms). Participants were asked to provide their response when the response screen appeared. A sound was played for 100 ms to 1500 ms after the participant’s response. Then, the participant was asked to estimate the delay between their response and the apparition of the sound, their decision confidence, and their agreement with the forced choice. The trial ended after feedback on the performance was given (correct: green tick; error: red cross).

The avoidance task consisted in detecting the orientation (left or right) of a target dot cloud while ignoring non-coherent dots, and avoiding this target cloud by pressing the key corresponding to one of the two directional arrows (right or left arrow) (see Fig. 1). Participants who failed to achieve 75% accuracy repeated the training phase.

This training was followed by a calibration phase, in which participants' performance at different levels of coherence was estimated using a double-staircase method, by fitting a psychometric curve to their data. The aim of this phase was to determine three levels of difficulty for the avoidance task (easy, medium and high).

Finally, the second training session involved performing the avoidance task followed by a temporal estimation task. The latter task involved participants estimating the interval between their response (left or right directional arrow) and a neutral sound that occurred shortly after this action. This interval estimation task was used to characterize a phenomenon known as “Temporal binding” (TB), which refers to the perceived *compression* of the temporal interval between a voluntary action and its external consequence^22^. This compression is often seen as an implicit marker of the sense of agency: a shorter perceived interval would indicate a higher sense of agency over the subsequent outcome (for a review, see). We computed TB as the difference between the perceived time and the actual delay between the button press and the sound, as routinely done in studies using the TB measure^26–28^. Note that, unlike the original study^13^, here we use only an *implicit* measure of SoA in order to mitigate drawbacks associated with explicit measures—such as posthoc rationalization effects or social desirability biases—, which could potentially impact the subsequent acceptability measure (for example, participants might report that the system is more acceptable when they previously reported that they felt more in control).

Once the first block (training and calibration) had been completed, participants carried out the testing phase.

#### Testing phase

The testing phase consisted of the avoidance task followed by the temporal estimation task (Fig. 1). A total of 432 trials were carried out, divided into 12 blocks of 36 trials. Of these, 216 trials assigned to each level of the experimental factors (Explicability, Automation and Difficulty—see below), and 144 trials assigned to each of the reliability conditions (low/medium/high). Two levels of difficulty (easy/hard), corresponding to the 2 levels estimated from the calibration phase, were used during the avoidance task.

##### Automation factor

Two types of trials were presented, in which the level of automation (free-choice vs. forced-choice) of the response was manipulated. In free-choice trials, participants chose the target arrow (left or right) according to the orientation of the cloud. In forced-choice trials, the computer preselected an arrow for the participant. Free-choice and forced-choice trials were randomly interleaved within each block. At the start of each trial, a brief sentence signaled the nature of the forthcoming condition (free trials: “You choose”; forced trials: “The system chooses”) to avoid anticipatory free responses from the participant (see^14,29,30^ for a similar procedure). Choice and system confidence were always aligned, with the system choosing the direction in which it was most confident.

##### Explicability factor

**Figure 2:**
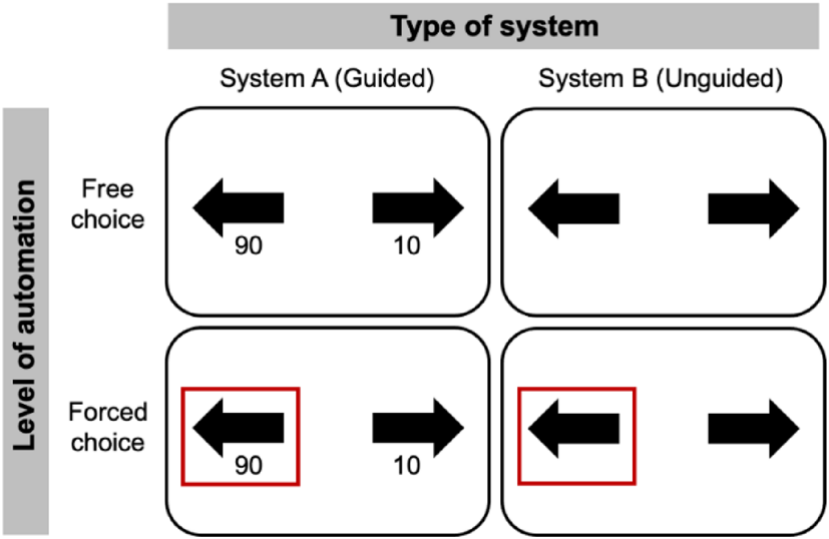
Illustration of the different types of response conditions. The free choice condition corresponds to trials in which the subject can choose the direction by herself. The forced choice condition corresponds to trials in which the subject must follow the choice of the system (indicated by a red square). One of the two systems (system A) guides the subject by returning its relative confidence (between 0 and 100) in each of the two possible answers. The second system returns nothing (Unguided).

Different decision support systems were implemented with different levels of reliability depending on the difficulty of the current trial. The reliability of the system was defined here by its performance, i.e., its rate of correct responses to the task (low/medium/high). In half of the trials, the system provided its *confidence* about its decision, while in the other half no confidence was provided. Trials with confidence were labelled “guided trials” whereas trials without confidence were labelled “unguided trials” (see Fig. 2). The confidence returned by system could range from 1 (the system was not confident in its decision) to 100 (the system was absolutely certain of its decision). Importantly, the confidence provided by the systems was calibrated according to the difficulty of the current trial and the reliability of the system. The assistance provided by the system was directly related to its reliability. Since participants produced an average of 100% correct responses on easy trials and 55% on difficult trials, the low-reliability system was therefore on average worse than participants, the medium-reliability system was on average as good as participants, and the high-reliability system was on average better than participants (see Suppl. Table 1).

The order of presentation of the reliability conditions was pseudo-randomly shuffled. To avoid system-related recency effects affecting interactions with other systems, all participants started with the 'medium' reliability system.

##### Time estimation task (“temporal binding”, TB)

The avoidance task was followed by a time estimation task, in which participants had to estimate the perceived time interval (1–1500 ms) between their response to the orientation of the cloud and a subsequent sound, among three possible responses (250 ms, 750 ms, and 1250 ms).

##### Decision confidence

Following the time estimation task, the participant was asked to indicate confidence in their performance on the avoidance task using a Likert scale ranging from 1 (low confidence: the participant thinks they did not avoid the cloud) to 8 (high confidence: the participant thinks they avoided the cloud). Depending on the type of trial, the participant was asked to judge either their own performance (free trial) or the performance of the system (forced trial). Following the confidence rating, a feedback (green tick or red cross for correct or incorrect responses) was given to the participant on their performance on the avoidance task (Fig. 2). This feedback was included so that the subject would be informed of their performance and that of the system, and could thus assess the reliability of the system's recommendations. It is important to note that TB was systematically measured *before* the feedback was delivered, and therefore could not be affected by the feedback itself.

##### Agreement with the forced choice

During the forced trials only, the participant was asked to indicate their agreement with the choice imposed by the system, using a Likert scale ranging from 1 (low agreement: the participant thinks the system is absolutely wrong) to 8 (high agreement: the participant thinks the system is absolutely right).

##### Acceptability measure

Finally, at the end of each block, participants rated the system's "acceptability" on two dimensions—usefulness and satisfaction—using a scale ranging from "not at all" to "completely". A total of 18 acceptability measures were collected per subject (one for each type of system encountered) and averaged to obtain a total acceptability score per subject (Sup. Table 3).

### Data analysis

Data were analyzed using the R software^31^ and the ezANOVA package^32^. For the Temporal Binding measure, the raw data were first filtered according to the interval reported by the participant for each interval category shown (250 ms, 750 ms and 1250 ms). Two exclusion criteria were used, based on a previous experiment with a similar paradigm^13^. First, in each participant, an average interval was calculated for each category and intervals that were two standard deviations above or below this average interval were considered outliers. We checked that no participant had performances containing more than 7.5% outliers (no participant was excluded on the basis of this criterion). To further test the reliability of the reported intervals, a second exclusion criterion was defined on the basis of the correlation between perceived and actual intervals for each interval category. Six participants were excluded due to low non-significant coefficients (R's < 0.2).

*Performance* to the avoidance task (% of correct responses and response times) and *agreement* score with the forced choice were analyzed using two 2 × 2 × 3 repeated-measures ANOVAs with explicability (guided vs. unguided), task difficulty (easy vs. high) and system reliability (low vs. medium vs. high) as within-participants factors, whereas *Temporal Binding*, *confidence* scores and *Response Times* (see Supplementary Results) were analyzed using two 2 × 2 × 2 × 3 repeated-measures ANOVAs with automation (free vs. forced-choice), explicability (guided vs. unguided), task difficulty (easy vs. hard) and system reliability (low vs. medium vs. high) as within-participants factors. Note that percentages of correct responses were only analyzed in the free-choice trials, where participants made an intentional choice. Main and interaction effects from all ANOVAs were further analyzed using Bonferroni-corrected post hoc pairwise comparisons. The normality assumption was met for the residuals of all dependent variables, with the exception of the confidence score (W = 0.9, *p* < 0.05). The data for this variable were therefore min–max normalized before analysis.

## Results

### Performance on the avoidance task—% correct responses

**Figure 3:**
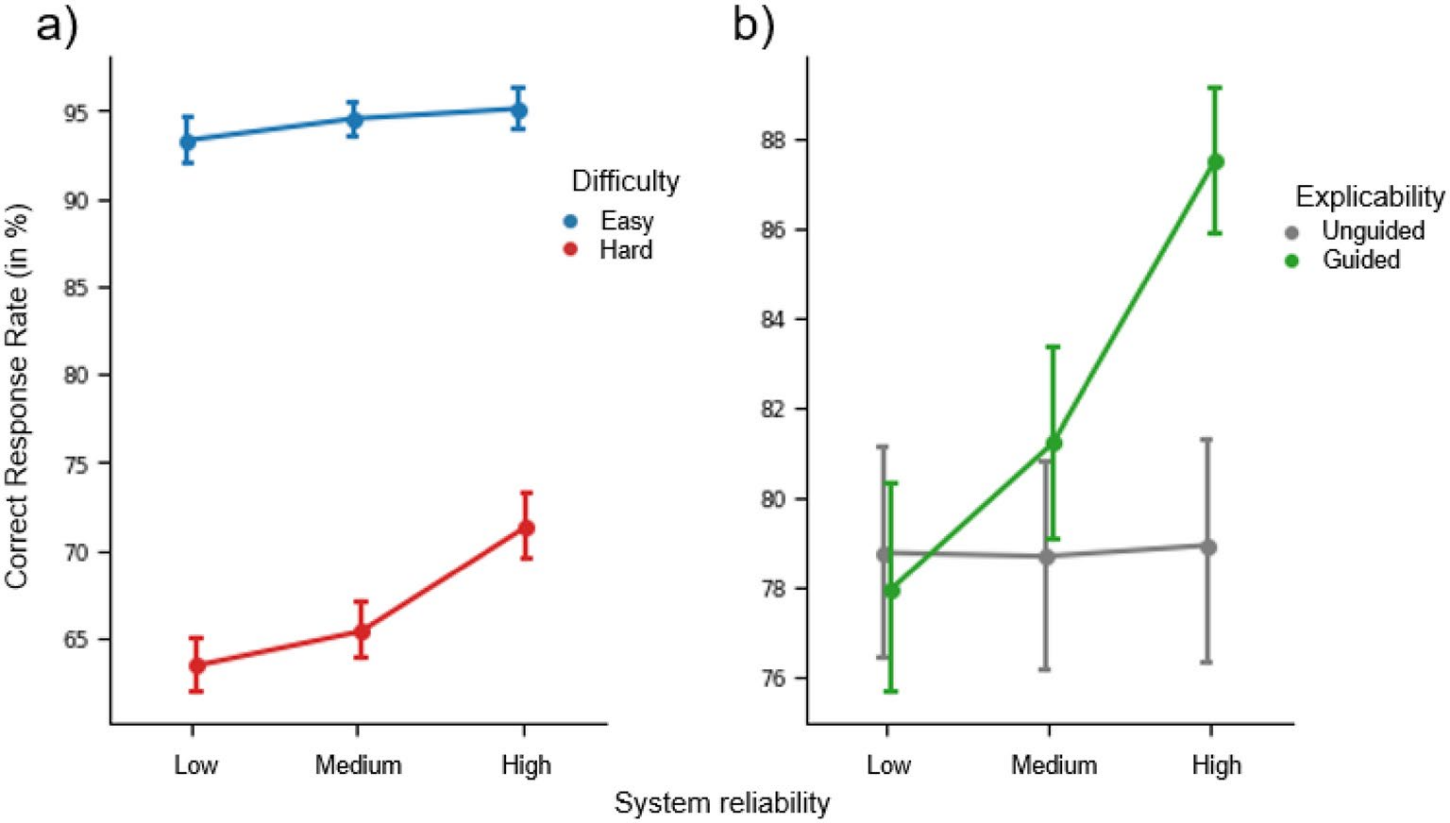
(**a**) Mean Correct Response Rate (in %) associated with the two levels of Difficulty (Easy vs. Hard) and System Reliability (Low vs. Medium vs. High) factors (left panel). (**b**) Mean Correct Response Rate (in %) associated with the two levels of Explicability (Guided vs. Unguided) and the system reliability (Low vs. Medium vs. High) factors (right panel). Error bars show ± 1 within-subject standard error of the mean (S.E.M).

The ANOVA revealed significant main effects of the explicability (mean correct rates: guided = 82.21 ± 17.44; unguided = 78.79 ± 19.68; F(1,33) = 13.32; *p* < 0.001, η_p_^2^ = 0.288), the reliability (mean correct rates: low-reliability = 78.34 ± 19.18; medium-reliability = 79.94 ± 18.42; high-reliability = 83.21 ± 18.14; F(1,33) = 12.43; *p* < 0.001, η_p_^2^ = 0.274) and the difficulty factors (mean correct rates: easy trials = 94.30 ± 5.20; hard trials = 66.7 ± 6.23; F(2,72) = 263,53; *p* < 0.001, η_p_^2^ = 0.889). A significant explicability-by-reliability interaction was also found (F(2,66) = 6.22; *p* = 0.003 ; η_p_^2^ = 0.159). Post hoc comparisons showed that the difference between guided and unguided trials is only significant for the high-reliability system (*p* < 0.001). A significant difficulty-by-reliability interaction was also found (F(2,66) = 5.02; *p* = 0.009; η_p_^2^ = 0.132, Fig. 3). Post hoc comparisons reveal that the difference between the systems is significant for the hard trials only (all *p* < 0.001). No other significant main or interaction effects were found (all p’s ≥ 0.05).

In summary, and as expected, the high-reliability system and the explainable system were the systems that most influenced participants to choose the correct direction (relative to the dot cloud). Participants also made more errors on hard trials when interacting with the low-reliability system.

### Temporal binding

**Figure 4:**
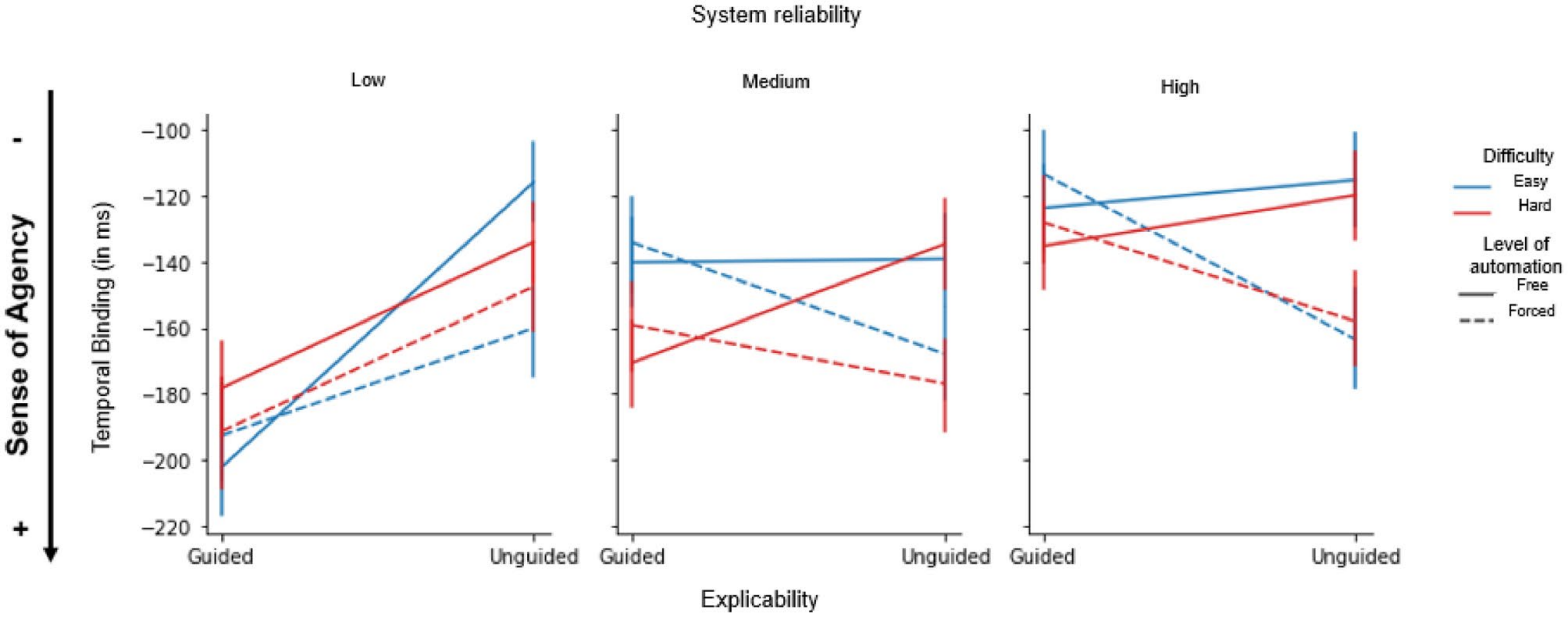
Average temporal binding associated with the automation (free vs. forced), explicability (guided vs. unguided), system reliability (low vs. medium vs. high) and difficulty factors (easy vs. high). Solid lines: easy trials; dashed lines: hard trials. Error bars show ± 1 within-subject standard error of the mean (S.E.M).

We found a marginal effect of the automation factor (forced: − 151.36 ± 3.88 vs. free: − 137.12 ± 3.78; F(1,33) = 4.02; *p* = 0.053; η_p_^2^ = 0.109) and a significant main effect of the reliability factor (F(1,33) = 9.02; *p* < 0.001; η_p_^2^ = 0.215) on the TB measure. Bonferroni-corrected post hoc comparisons revealed that the low-reliability system was associated with a stronger binding effect, i.e. with shorter action-outcome intervals indicating a higher SoA (low: − 158.98 ± 4.86 vs. medium: − 146.31 ± 4.67 vs. high: − 127.43 ± 4.55; all *p’s* < 0.003 except for low vs. medium: *p* = 0.3). A significant explicability-by-reliability interaction was also found (F(2,66) = 3.56; *p* = 0.034; η_p_^2^ = 0.097). Interestingly, system reliability had an effect on the guided trials only, with low-reliability system being associated with stronger binding effect (guided trials, low − 183.62 ± 7.37 vs. high: 120.95 ± 6.06; *p* < 0.001; Fig. 4). A significant difficulty-by-reliability interaction was also found (F(2,66) = 3.26; *p* = 0.045; η_p_^2^ = 0.090). Bonferroni-corrected post hoc comparisons showed that the difference was only significant between the low and high-reliability system (*p* < *0.01*) for the easy and hard trials (Easy trials, low: − 161.10 ± 6.89 < high: − 123.71 ± 6.44; Hard trials, low: − 156.56 ± 6.86 < high: − 131.15 ± 6.44). Another significant explicability-by-automation interaction was also found (F(2,66) = 14.59; *p* < 0.001; η_p_^2^ = 0.307). Bonferroni-corrected post hoc comparisons revealed that the difference between the free and forced trials was only significant for the unguided trials (Unguided trials, free: − 120.87; ± 5.29; forced: − 155.66 ± 5.29; *p* < 0.001; Fig. 4). No other significant main or interaction effects were found (all p’s ≥ 0.05).

In summary, participants' SoA was greater (i.e., perceived action-outcome intervals were shorter) when interacting with the low-reliability guided system (Fig. 4, left panel, red curves).

### Decision confidence

The repeated-measures ANOVA revealed significant main effects of the explicability (guided: 5.70 ± 0.07 vs. unguided: 5.57 ± 0.0.08; F(1,33) = 7.58; *p* = 0.01; η_p_^2^ = 0.187), the automation (free: 5.87 ± 0.08 vs. forced: 5.41 ± 0.07; F(2,66) = 47.19; *p* < 0.001; η_p_^2^ = 0.588), the difficulty (easy: 6.25 ± 0.07 vs. hard: 5.03 ± 0.08; F(2,66) = 84.47; *p* < 0.001; η_p_^2^ = 0.719) and the system reliability factors (low: 5.40 ± 0.08; medium: 5.63 ± 0.09; high: 5.89 ± 0.08; *p* < 0.001). The automation-by-reliability interaction was significant (F(2,66) = 32.82; *p* < 0.001; ηp^2^ = 0.499). Post hoc comparisons revealed that decision confidence was modulated by system reliability (low: 4.98 ± 0.04; medium: 5.43 ± 0.04; high: 5.80 ± 0.04) in forced trials only (all p’s < 0.0001). In free trials, the only discernible difference we found was between the medium and high-reliability systems (medium: 5.80 ± 0.04 vs. high: 5.97 ± 0.04, p = 0.04; all other comparisons, p > 0.05).

**Figure 5:**
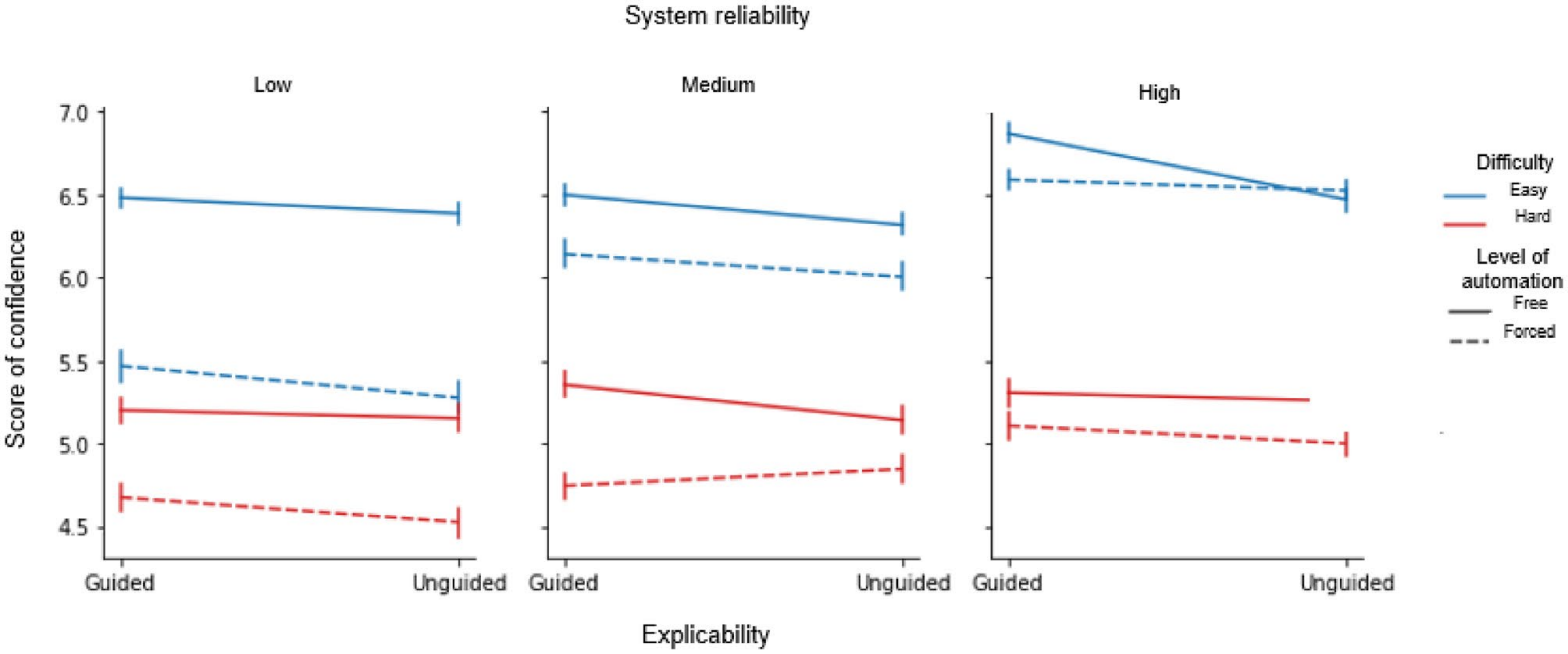
Average decision confidence scores associated with the automation (free vs. forced), explicability (guided vs. unguided), system reliability (low vs. medium vs. high) and difficulty factors (easy vs. high). Solid lines: easy trials; dashed lines: hard trials. Error bars show ± 1 within-subject standard error of the mean (S.E.M).

A significant difficulty-by-reliability interaction (F(2,66) = 17.85; *p* < 0.001; ηp^2^ = 0.351) was also found. Post hoc comparisons revealed that decision confidence was modulated by system reliability (low: 5.90 ± 0.04; medium: 6.23 ± 0.04; high: 6.61 ± 0.03) in easy trials (all *p* < 0.0001) and to a lesser extent in hard trials (low: 4.88 ± 0.04 vs high: 5.16 ± 0.04, *p* = 0.019; all other comparisons, *p* > 0.05). The explicability-by-automation-by-reliability triple interaction was also significant (F(2,66) = 3.58; p = 0.034; ηp^2^ = 0.098). Post-hoc comparisons revealed that decision confidence was modulated by the level of automation (free > forced) and by system reliability (free vs forced, low: *p* < 0.001; medium: *p* < 0.05; high: *p* > 0.05), independently of the explicability factor. The difficulty-by-automation-by-reliability triple interaction (F(2,66) = 11.67; *p* < 0.001; ηp^2^ = 0.261) was also significant. Post-hoc comparisons showed that the difference between the two levels of automation (free > forced) was reduced when system reliability increased, independently of task difficulty (free vs forced, low: *p* < 0.001; medium: *p* < 0.001; high: *p* > 0.05). The explicability-by-automation-by-reliability-by-difficulty quadruple interaction was significant (F(2,66) = 3.73; *p* = 0.029; ηp^2^ = 0.102; Fig. 5).

Decomposition of the 4-way interaction using post hoc tests revealed that participants were more confident in their decision on free trials when interacting with the low-reliability system—i.e. when they had to make a free choice in a condition where the system made more errors (p < 0.001). This difference disappeared as reliability increased (all other comparisons, *p* > 0.05).

Together, these results show that all four factors tested influenced decision confidence. As expected, participants were more confident in their decision when interacting with the high-reliability system. In these high-reliability conditions, the influence of automation levels (free > forced) was also the smallest, and the influence of task difficulty (easy > difficult trials) was, in contrast, the largest.

### Agreement with forced trials

The repeated-measures ANOVA revealed significant main effects of task difficulty (easy: 6.09 ± 2.32 vs. hard: 5.15 ± 2.12; F(2,66) = 84.47; p < 0.001 ; η_p_^2^ = 0.719) and system reliability (low: 4.94 ± 2.43 vs. medium: 5.65 ± 2.26 vs. high: 6.28 ± 1.89; p < 0.001). The explicability-by-reliability interaction was significant (F(2,66) = 4.93; p = 0.01; ηp^2^ = 0.130). Post hoc comparisons revealed that explicability influenced the agreement score (guided > unguided), specifically when participants interacted with the high-reliability system (p = 0.05). The difficulty-by-reliability interaction was also significant (F(2,66) = 32.72; p < 0.001; ηp^2^ = 0.498). Post hoc comparisons showed that difficulty influenced the agreement score (easy > hard), specifically when participants interacted with medium- and high-reliability systems (easy vs hard: low reliability: *p* = 0.1; medium- and high-reliability: *p* < 0.001). The explicability-by-difficulty-by-reliability triple interaction was also significant (F(2,66) = 5.73; *p* = 0.006; ηp^2^ = 0.207). Post-hoc comparisons revealed that agreement scores were influenced by system reliability (low < medium < high), specifically when the system communicated its confidence (guided trials) in easy (all *p’s* < 0.001) and hard trials (*p* < 0.01). When the system did not communicate its confidence (unguided trials), the influence of system reliability was observed in easy trials (all *p* < 0.001) and between low- and high-reliability systems in hard trials (low vs high: p = 0.001 all other comparisons, p > 0.05) (see Sup Fig. 2). No other significant main or interaction effects were found (all p’s > 0.05).

In summary, all three factors influenced agreement with system choice. As expected, participants agreed more with the high-reliability system in the easy and guided trials. However, when the system did not communicate confidence (unguided trials), the influence of the reliability factor on agreement was primarily observed in the easy trials.

### Acceptability scales

**Figure 6:**
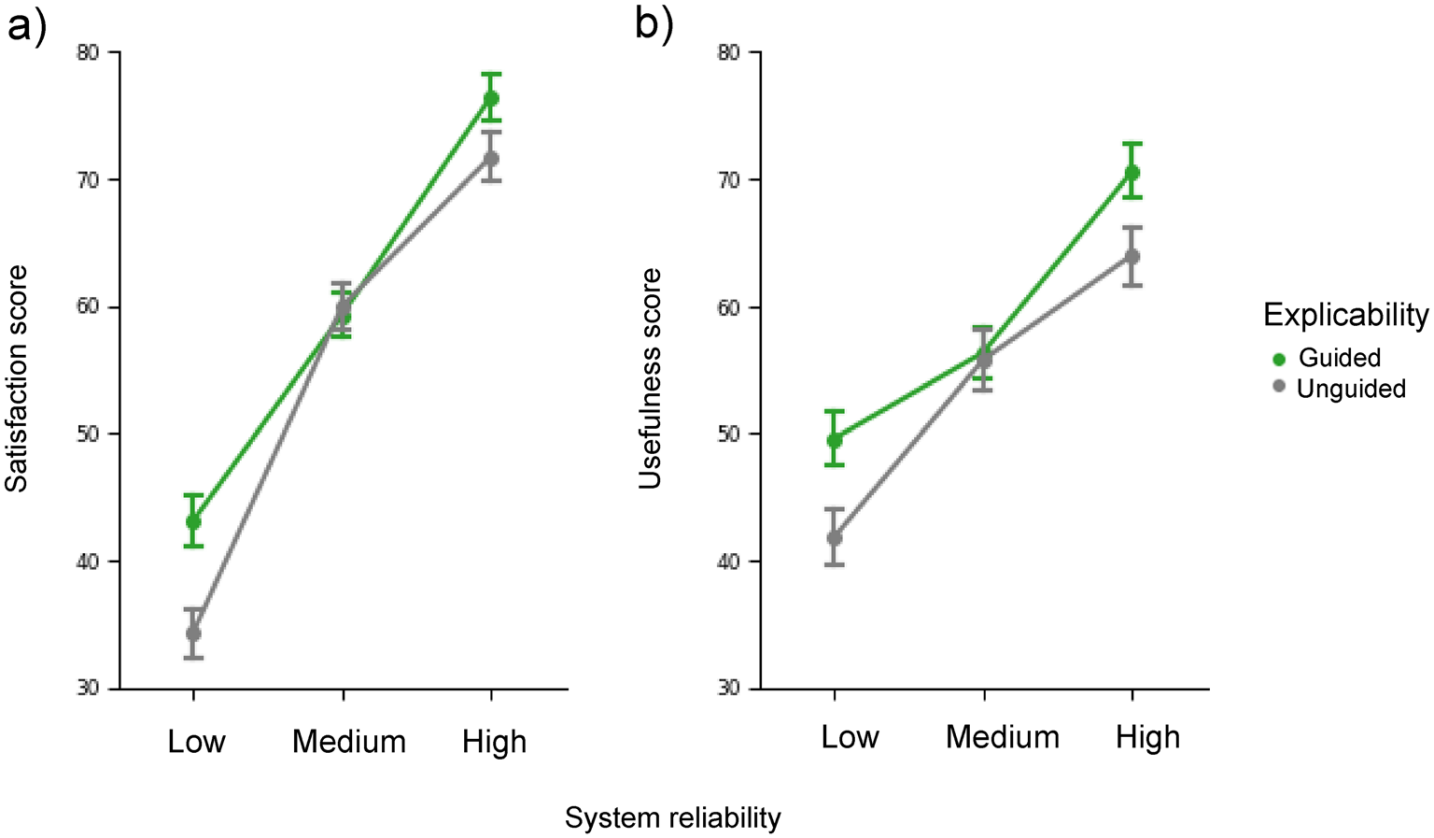
(**a**) Mean Satisfaction Score associated with the two levels of explicability (Guided vs. No Guided) and system reliability (Low vs. Medium vs. High) factors (left panel). (**b**) Mean Usefulness Score associated with the two levels of explicability (Guided vs. No Guided) and the system reliability (Low vs. Medium vs. High) factors (right panel). Error bars show ± 1 within-subject standard error of the mean (S.E.M).

For the Satisfaction score, the repeated-measures ANOVA revealed main effects of the explicability (guided: 59.62 ± 2.01 vs. unguided: 55.33 ± 1.89, F(1,33) = 6.75; *p* = 0.014; η_p_^2^ = 0.170) and system reliability factors (low: 38.70 ± 2.50 vs. medium: 38.69 ± 2.23 vs. high: 74.10 ± 2.08; F(1,33) = 175.51; *p* < 0.001; η_p_^2^ = 0.842). In addition, the explicability-by-reliability interaction was significant (F(2,66) = 4.41; *p* = 0.016; ηp^2^ = 0.118; Fig. 6a). Post-hoc analyses revealed that explicability (guided > unguided) influenced satisfaction only when participants interacted with the low-reliability system (*p* < 0.001). For the Usefulness score, the repeated-measures ANOVA revealed main effects of explicability (guided: 58.84 ± 1.87; unguided: 54.01 ± 1.94; F(1,33) = 9.39; *p* = 0.004; ηp^2^ = 0.221) and system reliability (low: 45.12 ± 1.87; medium: 55.20 ± 2.23; high: 66.95 ± 2.10; F(1,33) = 57.70; *p* < 0.001; ηp^2^ = 0.636). (Fig. 6b).

In summary, and as expected, participants found the guided and more reliable system more acceptable—both on the usefulness and satisfaction dimensions.

## Discussion

It is now accepted that providing assistance to human operators can improve their performance without altering their sense of control (SoA)^10,13,27^. Thus, when the assistance system is effectively calibrated, both in terms of objective performance and metacognitive evaluation, the operator's SoA naturally benefits from the cues communicated by the system^13^. One reason for this could be that a well-calibrated system allows for a better allocation of resources in the action or task. In this study, we sought to test this hypothesis further by determining whether, and to what extent, the SoA of human operators interacting with an assistance system is modulated when the reliability of the assistance varies. To do so, we selectively changed the level of performance (or reliability) of the assistance system and measured the influence of this change on the SoA and acceptability of human participants.

### Reliability and sense of control

We first hypothesized that system reliability would modulate participants' SoA, and specifically the amount of control used to perform the task (see^13^ for the relationship between SoA and control used). In particular, we predicted that the lower the reliability of the system, the higher the SoA would be, as low reliability requires the operator to commit more cognitive resources to the action to maintain control. We first observed that participants were able to discriminate between the different types of systems they interacted with. Thus, participants were overall more confident in their own decision (Fig. 5) and more often disagreed with the system choice when interacting with low-reliability systems. As expected, participants' SoA was modulated by system reliability. Specifically, participants' SoA was higher when they interacted with the low-reliability system. Our results are consistent with previous studies investigating "attentional allocation” strategies during human–machine interaction with imperfect assistance^33–35^. These studies showed that when participants interact with a less reliable system, they commit more cognitive control to the task. Variations in our implicit measure of control (temporal binding) appear to reflect these variations in the amount of control allocated to the current task as a function of the reliability of the assistance system.

### Explicability, automation and sense of control

Our second hypothesis was that communicating the system's confidence in the best decision to be made (explicability) would enable agents to better regulate the allocation of their control resources in the current task. We observed an interaction between the explicability and reliability factors: participants' SoA, as measured by temporal binding, was primarily impacted by the system's confidence in the *guided* condition. In other words, participants' SoA was highest when the system's reliability was low, but only when the system returned information about confidence in its decision; conversely, SoA was lowest when no explanation was provided. Taken together, these results suggest that the metacognitive information provided by the system would help select the optimal policy for cognitive control engagement.

Cognitive control^36,37^ engagement is known to depend on detection and awareness of variations in task demands. It is likely that the metacognitive information provided by the system helps participants become aware of these variations in task demands and contributes to a more flexible engagement of cognitive control. Interestingly, in the presence of metacognitive information, the level of automation did not affect the level of SoA, such that participants experienced similar control in the free and forced conditions. An opposite pattern of results was observed in the *unguided* condition. Specifically, we found that SoA was highest when participants were forced to choose the system. In other words, in the absence of metacognitive information about the system's confidence in its own performance, cognitive control regulation becomes more difficult, and the participant's SoA depends primarily on the assistance provided by the automated system.

Together, these results suggest that participants' SoA can be influenced by different sources of information depending on their reliability, but also their availability^3,38^. When the system guides the decision (via metacognitive information), its reliability plays a major role in decision making and in the formation of the SoA (Fig. 4). This positive influence of explicability on participants' SoA suggests that the explicability of the system helps to proactively optimize the resources to be committed to making the right decision^39^. In contrast, when the system does not guide (no presence of metacognitive information), the reliability of the system does not influence the participant’s SoA, which therefore relies on other types of information, such as the level of automation of the task (Fig. 4)^11, 13^.

These results are consistent with recent studies that showed that making a system more "explainable", i.e., making it less opaque (via a bonus or confidence in its decision), benefits to the SoA of participants interacting with it^13^. Our results further suggest that the confidence communicated by the assistance system can be used by the participant as a metacognitive signal to adjust the amount of resources to invest in the current task (metacognitive control^40^).

### Explicability and acceptability

We hypothesized that a decrease in SoA, caused by a decrease in system reliability, would be associated with a decrease in system acceptability^11,13^. Our measures of acceptability, represented by the usefulness and satisfaction scores, were indeed positively influenced by system explicability. Critically, both guided and unguided systems had the same levels of performance, yet the guided system was generally perceived more favourably. As already suggested in the literature^11,13^, improving the explicability of systems, by making them more intelligible to humans, improves their usefulness, and the communication of metacognitive information could contribute to making the automated system more cooperative. Interestingly, our measures of acceptability were also positively influenced by system reliability: participants felt greater overall acceptability for the high reliability system (Fig. 5). As noted above, this result suggests that the measures of control and acceptability used in this study do not index the same thing. While the temporal binding measure would be sensitive to the demands of the task at hand, and would therefore reflect a proxy for the amount of resources the subjects believe they should invest in the task, acceptability would be directly related to the experience of control felt by operators when interacting with the system^41^.

## Conclusions and perspectives

Our results show that participants' SoA can be influenced by different sources of information. Metacognitive information, when provided by the system during the decision (i.e., in guided conditions), appear to play an important role in decision making and SoA formation, by allowing the participant to engage the appropriate control resources in the task at hand. When the system does not deliver metacognitive information (i.e., unguided conditions), participants rely on other types of information, such as the level and nature of task automation. Our results presuppose a strong link between the resource engaged in the action and the temporal binding measure (used as a proxy for the participant’s control experience). Interestingly, recent studies^42,43^ have linked cognitive resources to response times. Therefore, we examined response times, and a significant effect of system reliability was observed (see Supplementary Analysis). However, the results obtained must be taken with caution, as the protocol used was not specifically designed to analyze response times—i.e., we did not ask participants to respond as quickly as possible. To further test whether interaction with a more or less opaque (or reliable) decision support system leads to different management of attentional resources, future research could probe more directly the control resources engaged by the participant during the task, either using behavioral measures (such as the "control used" scale used in^13, 41^) or using objective metrics such as eye-tracking. Time spent on a particular area of a screen is indeed traditionally considered an indication of difficulty in extracting relevant information from that area^44,45^. Eye-tracking could thus help determine the participant's focus of attention during the task, as well as the duration of this focus, and identify the moment when the participant becomes aware of the information returned by the system. Eye-tracking, as an indirect measure of attentional resource management, would usefully complement the behavioral measures employed in this paradigm.

Another potential limitation of the study is the apparent contradiction between measures of confidence and SoA: participants report greater confidence in their decisions in unguided free-choice trials compared with forced-choice trials, while showing the lowest SoA in these conditions. We believe, however, that this contradiction is only apparent and stems from the fact that confidence and SoA do not measure the same thing: while SoA measures, or expresses, causal responsibility for the outcome of a decision, confidence instead estimates the subjective probability of being correct in one's decision^46^. Our tentative explanation is that, under conditions of (unguided) free choice, participants are more likely to monitor their internal performance-related signals rather than focus on external recommendations, and therefore tend to express greater confidence in their choice as a result.

Making a system more explainable (or intelligible to the human operator) in its choices may reduce the physical and cognitive distance between the system and the operator, a distance that can adversely affect both performance and user experience in human–computer interactions^47^. Our results support the idea that the subjective experience of control in human–machine interaction could be used as a guideline to make systems more agentic and acceptable^11,13^.

### Supplementary Information

Supplementary Information.

## Data Availability

The datasets generated and/or analysed as part of this study are available in the “*Reliability and agency data”* repository, https://osf.io/syk79.
