# Supplementary material for: The reliability of assistance systems modulates the sense of control and acceptability of human operators
Source: Sci Rep. 2023 Sep 2;13:14410. doi: 10.1038/s41598-023-41253-8 (PMC10475027; doi:10.1038/s41598-023-41253-8)
Supplement: Supplementary file 1 — Supplementary Information. [file 41598_2023_41253_MOESM1_ESM.docx]

# SUPPLEMENTARY MATERIAL: The reliability of assistance systems modulates the sense of control and acceptability of human operators

Quentin Vantrepotte, Valérian Chambon, Bruno Berberian

#
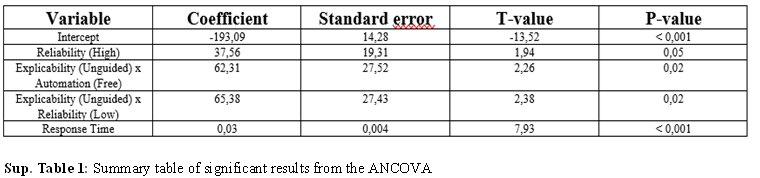
Methods: Supplementary Tables

**Supp. Table 2:** Percentage of correct rate for the subjects and the system associated with the Level of Difficulty


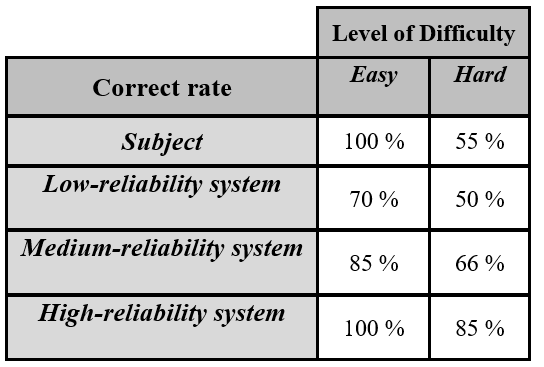

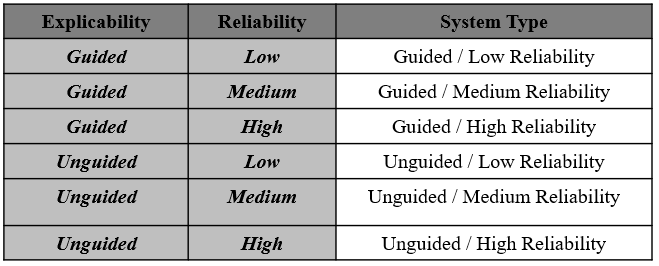


**Supp. Table 3**: Illustration of the different types of systems associated with Explicability and Reliability

# Supplementary Results

**Response time**

The repeated-measures ANOVA revealed significant main effects of the explicability (guided: 1015 ± 380; unguided: 1015 ± 395; F(1,33) = 34.09; *p =* 0.001; η_p_² = 0.508), the difficulty (easy: 817 ± 387 vs. hard: 1075 ± 390; F(1,33) = 47.23; *p* < 0.001 ; η_p_² = 0.589) and the system reliability factors (low: 995 ± 325; medium: 997 ± 333; high: 846 ± 300; *p* < 0.001) (**Sup. Figure 1**).

The explicability-by-difficulty interaction was significant (F(1,33) = 10.84; *p* = 0.003; ηp² = 0.239). Post hoc comparisons indicate that response times were not modulated by explicability (guided > unguided), regardless of difficulty (all *p’s* < 0.05). The explicability-by-reliability interaction was significant (F(2,66) = 5.98; *p* = 0.004; ηp² = 0.153). Post hoc comparisons revealed that the difference between the two levels of explicability (guided > unguided) was reduced when system reliability increased (low: *p* < 0.001; medium: *p* < 0.05; high: *p* > 0.05). The difficult-by-reliability interaction was significant (F(2,66) = 4.22; *p* = 0.019; ηp² = 0.113). Post hoc comparisons revealed that the difference between the two levels of difficulty (easy < hard) was higher when system reliability increased (all *p’s* < 0.001). The explicability-by-automation interaction was also significant (F(1,33) = 15.05; *p* < 0.001; ηp² = 0.313). Post hoc comparisons show that the difference between the two levels of automation (forced > free) was only discernible in unguided trials (unguided: *p =* 0.001; guided: *p* > 0.05). The difficulty-by-automation interaction was significant (F(1,33) = 9.72; *p* = 0.004; ηp² = 0.227). Post hoc comparisons revealed that the difference between the two levels of automation (forced > free) was only significant in easy trials (easy: *p =* 0.002; hard: *p* > 0.05). The reliability-by-automation interaction was significant (F(2,66) = 13.50; *p* < 0.001; ηp² = 0.290). Post hoc comparisons revealed no significant difference between levels of automation and system reliability (all *p’s >* 0.05).

The explicability-by-difficulty-by-reliability triple interaction was significant (F(2,66) = 3.23; p = 0.046; ηp² = 0.089). Post-hoc comparisons revealed that response time was modulated by explicability (guided > unguided), specifically when interacting with the low-reliability system (easy: *p* = 0.001; hard: *p* = 0.002) and the medium-reliability system in hard trials (*p* < 0.001). The automation-by-difficulty-by-reliability triple interaction was significant as well (F(2,66) = 4.02; p = 0.023; ηp² = 0.109). Post-hoc comparisons revealed that response time was modulated by the level of automation (forced > free), but only in easy trials and specifically when interacting with the low-reliability (*p* = 0.002) and medium-reliability (*p* = 0.006) systems.

Finally, the automation-by-difficulty-by-reliability-by-explicability quadruple interaction was significant (F(2,66) = 6.38; p = 0.003; ηp² = 0.162). Decomposition of the 4-way interaction using post hoc tests revealed that participants were slower in their response in guided trials mostly when interacting with the low-reliability and the medium-reliability systems, and only in hard trials (p < 0.05). No other comparisons revealed significant differences (p > 0.05).

In summary, all four factors influenced response times, and this influence was potentiated by system reliability.

**Supp. Figure 1:** Average Response Time associated with the Automation (Free vs. Forced), Explicability (Guided vs. Unguided), System Reliability (Low vs. Medium vs. High) and Difficulty (Easy vs. High) factors. Error bars show +/−1 within-subject standard error of the mean (S.E.M).


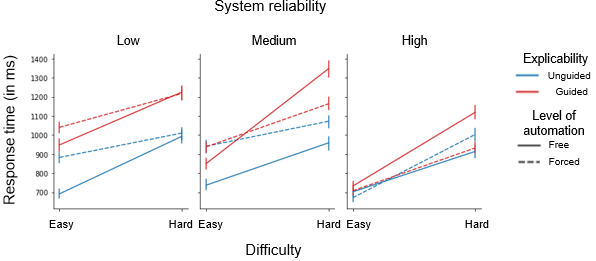


**Agreement with forced trials**


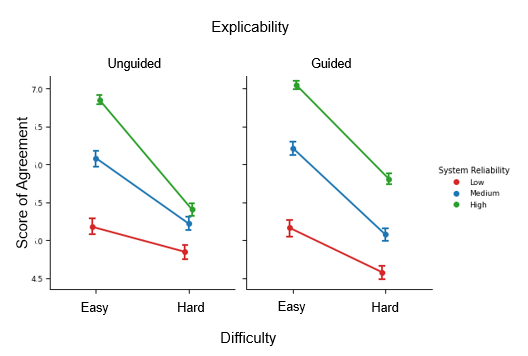


**Supp. Figure 2**: Mean agreement scores associated with the Explicability (Guided vs. Unguided), System Reliability (Low vs. Medium vs. High) and Difficulty factors (Easy vs. High). Solid lines: easy trials; Dashed lines: hard trials. Error bars show +/−1 within-subject standard error of the mean (S.E.M).

## Bonferroni-corrected posthoc analyses

### % Correct Response

**Table of Bonferroni Analyses** **- Reliability**

| Conditions tested | Estimate | Standard Error | DF | t-ratio | P-value |
| --- | --- | --- | --- | --- | --- |
| Low Reliability - Medium Reliability | -1.59 | 0.949 | 33 | -1.678 | 0.1028 |
| Low Reliability - High Reliability | **-4.86** | **1.031** | **33** | **-4.714** | **<.0001** |
| Medium Reliability - High Reliability | **-3.27** | **1.000** | **33** | **-3.268** | **0.0025** |

**Table of Bonferroni Analyses – Explicability x Reliability**

| Conditions compared | Estimate | Standard Error | DF | t-ratio | P-value |
| --- | --- | --- | --- | --- | --- |
| Guided Low Reliability - Unguided Low Reliability | -0.8170 | 1.710 | 33 | -0.478 | 0.9966 |
| Guided Low Reliability - Guided Medium Reliability | -3.2680 | 1.882 | 33 | -1.737 | 0.5186 |
| Guided Low Reliability - Unguided Medium Reliability | -0.7353 | 1.614 | 33 | -0.456 | 0.9973 |
| Guided Low Reliability - Guided High Reliability | **-9.5588** | **1.953** | **33** | **-4.896** | **0.0003** |
| Guided Low Reliability - Unguided High Reliability | -0.9804 | 1.675 | 33 | -0.585 | 0.9913 |
| Unguided Low Reliability - Guided Medium Reliability | -2.4510 | 0.929 | 33 | -2.638 | 0.1163 |
| Unguided Low Reliability - Unguided Medium Reliability | 0.0817 | 1.324 | 33 | 0.062 | 1.0000 |
| Unguided Low Reliability - Guided High Reliability | **-8.7418** | **1.470** | **33** | **-5.946** | **<.0001** |
| Unguided Low Reliability - Unguided High Reliability | -0.1634 | 1.786 | 33 | -0.091 | 1.0000 |
| Guided Medium Reliability - Unguided Medium Reliability | 2.5327 | 1.494 | 33 | 1.695 | 0.5447 |
| Guided Medium Reliability - Guided High Reliability | **-6.2908** | **1.428** | **33** | **-4.405** | **0.0014** |
| Guided Medium Reliability - Unguided High Reliability | 2.2876 | 1.757 | 33 | 1.302 | 0.7819 |
| Unguided Medium Reliability - Guided High Reliability | **-8.8235** | **1.834** | **33** | **-4.812** | **0.0004** |
| Unguided Medium Reliability - Unguided High Reliability | -0.2451 | 1.592 | 33 | -0.154 | 1.0000 |
| Guided High Reliability - Unguided High Reliability | **8.5784** | **2.189** | **33** | **3.919** | **0.0052** |

### Temporal Binding

**Table of Bonferroni Analyses - Reliability**

| Conditions compared | Estimate | Standard Error | DF | t.ratio | p.value |
| --- | --- | --- | --- | --- | --- |
| Medium Reliability - Low Reliability | 12.5 | 8.32 | 33 | 1.498 | 0.3047 |
| Medium Reliability - High Reliability | **-19.9** | **5.16** | **33** | **-3.864** | **0.0014** |
| Low Reliability - High Reliability | **-32.4** | **9.04** | **33** | **-3.584** | **0.0030** |

**Table of Bonferroni Analyses - Explicability x Automation**

| Conditions compared | Estimate | STANDARD ERROR | DF | t.ratio | p.value |
| --- | --- | --- | --- | --- | --- |
| Unguided Forced - Guided Forced | -9.80 | 7.23 | 33 | -1.355 | 0.5354 |
| Unguided Forced - Unguided Free | **-35.07** | **9.60** | **33** | **-3.653** | **0.0047** |
| Unguided Forced - Guided Free | -4.58 | 8.36 | 33 | -0.547 | 0.9466 |
| Guided Forced - Unguided Free | -25.27 | 10.23 | 33 | -2.469 | 0.0839 |
| Guided Forced - Guided Free | 5.22 | 8.62 | 33 | 0.606 | 0.9294 |
| Unguided Free - Guided Free | **30.49** | **8.21** | **33** | **3.716** | **0.0040** |

**Table of Bonferroni Analyses - Explicability x Reliability**

| Conditions compared | Estimate | Standard Error | DF | t.ratio | p.value |
| --- | --- | --- | --- | --- | --- |
| Unguided Medium Reliability - Guided Medium Reliability | -4.318 | 9.16 | 33 | -0.471 | 0.9968 |
| Unguided Medium Reliability - Unguided Low Reliability | -14.633 | 9.71 | 33 | -1.506 | 0.6627 |
| Unguided Medium Reliability - Guided Low Reliability | 35.257 | 13.94 | 33 | 2.528 | 0.1451 |
| Unguided Medium Reliability - Unguided High Reliability | -14.821 | 10.78 | 33 | -1.375 | 0.7413 |
| Unguided Medium Reliability - Guided High Reliability | -29.358 | 14.73 | 33 | -1.993 | 0.3676 |
| Guided Medium Reliability - Unguided Low Reliability | -10.315 | 7.39 | 33 | -1.395 | 0.7294 |
| Guided Medium Reliability - Guided Low Reliability | 39.574 | 16.26 | 33 | 2.434 | 0.1741 |
| Guided Medium Reliability - Unguided High Reliability | -10.503 | 12.60 | 33 | -0.833 | 0.9590 |
| Guided Medium Reliability - Guided High Reliability | -25.040 | 11.71 | 33 | -2.139 | 0.2931 |
| Unguided Low Reliability - Guided Low Reliability | **49.890** | **15.71** | **33** | **3.176** | **0.0349** |
| Unguided Low Reliability - Unguided High Reliability | -0.188 | 11.80 | 33 | -0.016 | 1.0000 |
| Unguided Low Reliability - Guided High Reliability | -14.725 | 14.10 | 33 | -1.044 | 0.8993 |
| Guided Low Reliability - Unguided High Reliability | **-50.078** | **7.75** | **33** | **-6.458** | **<.0001** |
| Guided Low Reliability - Guided High Reliability | **-64.614** | **24.78** | **33** | **-8.457** | **<.0001** |
| Unguided High Reliability - Guided High Reliability | -14.537 | 20.95 | 33 | -0.694 | 0.9814 |

**Table of Bonferroni Analyses - Difficulty x Reliability**

| Conditions compared | Estimate | STANDARD ERROR | DF | t.ratio | p.value |
| --- | --- | --- | --- | --- | --- |
| Easy Medium Reliability - Hard Medium Reliability | 15.10 | 9.39 | 33 | 1.607 | 0.5996 |
| Easy Medium Reliability - Hard Medium Reliability | 15.10 | 9.39 | 33 | 1.607 | 0.5996 |
| Easy Medium Reliability - Easy Low Reliability | 22.18 | 9.90 | 33 | 2.240 | 0.2473 |
| Easy Medium Reliability - Hard Low Reliability | 17.86 | 12.53 | 33 | 1.425 | 0.7117 |
| Easy Medium Reliability - Easy High Reliability | -14.49 | 6.72 | 33 | -2.158 | 0.2843 |
| Easy Medium Reliability - Hard High Reliability | -10.27 | 9.69 | 33 | -1.060 | 0.8934 |
| Hard Medium Reliability - Easy Low Reliability | 7.08 | 9.96 | 33 | 0.711 | 0.9793 |
| Hard Medium Reliability - Hard Low Reliability | 2.76 | 8.52 | 33 | 0.324 | 0.9995 |
| Hard Medium Reliability - Easy High Reliability | **-29.59** | **9.67** | **33** | **-3.059** | **0.0460** |
| Hard Medium Reliability - Hard High Reliability | **-25.37** | **6.20** | **33** | **-4.090** | **0.0033** |
| Easy Low Reliability - Hard Low Reliability | -4.32 | 7.84 | 33 | -0.551 | 0.9935 |
| Easy Low Reliability - Easy High Reliability | **-36.67** | **9.44** | **33** | **-3.883** | **0.0057** |
| Easy Low Reliability - Hard High Reliability | **-32.45** | **10.29** | **33** | **-3.154** | **0.0368** |
| Hard Low Reliability - Easy High Reliability | -32.35 | 13.06 | 33 | -2.477 | 0.1605 |
| Hard Low Reliability - Hard High Reliability | -28.13 | 9.95 | 33 | -2.827 | 0.0779 |
| Easy High Reliability - Hard High Reliability | 4.22 | 8.74 | 33 | 0.483 | 0.9965 |

### Confidence score

**Table of Bonferroni Analyses - Reliability**

| Conditions compared | Estimate | Standard Error | DF | t-ratio | P-value |
| --- | --- | --- | --- | --- | --- |
| Medium Reliability - Low Reliability | **0.224** | **0.0521** | **33** | **4.304** | **0.0004** |
| Medium Reliability - High Reliability | **-0.264** | **0.0487** | **33** | **-5.422** | **<.0001** |
| Low Reliability - High Reliability | -0.488 | 0.0736 | 33 | -6.629 | **<.0001** |

**Table of Bonferroni Analyses – Automation x Reliability**

| Conditions compared | Estimate | Standard Error | DF | t-ratio | P-value |
| --- | --- | --- | --- | --- | --- |
| Forced Medium Reliability - Free Medium Reliability | **-0.39734** | **0.0745** | **33** | **-5.331** | **0.0001** |
| Forced Medium Reliability - Forced Low Reliability | **0.43422** | **0.0749** | **33** | **5.795** | **<.0001** |
| Forced Medium Reliability - Free Low Reliability | **-0.38330** | **0.0767** | **33** | **-4.999** | **0.0002** |
| Forced Medium Reliability - Forced High Reliability | **-0.37404** | **0.0650** | **33** | **-5.756** | **<.0001** |
| Forced Medium Reliability - Free High Reliability | **-0.55099** | **0.0829** | **33** | **-6.650** | **<.0001** |
| Free Medium Reliability - Forced Low Reliability | **0.83156** | **0.1151** | **33** | **7.227** | **<.0001** |
| Free Medium Reliability - Free Low Reliability | 0.01404 | 0.0501 | 33 | 0.280 | 0.9997 |
| Free Medium Reliability - Forced High Reliability | 0.02331 | 0.0717 | 33 | 0.325 | 0.9995 |
| Free Medium Reliability - Free High Reliability | -0.15365 | 0.0504 | 33 | -3.048 | 0.0472 |
| Forced Low Reliability - Free Low Reliability | **-0.81753** | **0.1041** | **33** | **-7.854** | **<.0001** |
| Forced Low Reliability - Forced High Reliability | **-0.80826** | **0.1125** | **33** | **-7.183** | **<.0001** |
| Forced Low Reliability - Free High Reliability | **-0.98521** | **0.1231** | **33** | **-8.001** | **<.0001** |
| Free Low Reliability - Forced High Reliability | 0.00927 | 0.0728 | 33 | 0.127 | 1.0000 |
| Free Low Reliability - Free High Reliability | -0.16769 | 0.0560 | 33 | -2.994 | 0.0535 |
| Forced High Reliability - Free High Reliability | **-0.17695** | **0.0612** | **33** | **-2.890** | **0.0677** |

**Table of Bonferroni Analyses – Difficulty x Reliability**

| Conditions compared | Estimate | Standard Error | DF | t-ratio | P-value |
| --- | --- | --- | --- | --- | --- |
| Easy Medium Reliability - Hard Medium Reliability | **1.219** | **0.1267** | **33** | **9.623** | **<.0001** |
| Easy Medium Reliability - Easy Low Reliability | **0.325** | **0.0576** | **33** | **5.643** | **<.0001** |
| Easy Medium Reliability - Hard Low Reliability | **1.343** | **0.1427** | **33** | **9.410** | **<.0001** |
| Easy Medium Reliability - Easy High Reliability | **-0.371** | **0.0570** | **33** | **-6.512** | **<.0001** |
| Easy Medium Reliability - Hard High Reliability | **1.063** | **0.1479** | **33** | **7.186** | **<.0001** |
| Hard Medium Reliability - Easy Low Reliability | **-0.894** | **0.1330** | **33** | **-6.726** | **<.0001** |
| Hard Medium Reliability - Hard Low Reliability | 0.123 | 0.0632 | 33 | 1.952 | 0.3906 |
| Hard Medium Reliability - Easy High Reliability | **-1.591** | **0.1423** | **33** | **-11.176** | **<.0001** |
| Hard Medium Reliability - Hard High Reliability | -0.156 | 0.0607 | 33 | -2.574 | 0.1326 |
| Easy Low Reliability - Hard Low Reliability | **1.018** | **0.1358** | **33** | **7.494** | **<.0001** |
| Easy Low Reliability - Easy High Reliability | **-0.696** | **0.0859** | **33** | **-8.103** | **<.0001** |
| Easy Low Reliability - Hard High Reliability | **0.738** | **0.1558** | **33** | **4.738** | **0.0005** |
| Hard Low Reliability - Easy High Reliability | **-1.714** | **0.1593** | **33** | **-10.761** | **<.0001** |
| Hard Low Reliability - Hard High Reliability | **-0.280** | **0.0815** | **33** | **-3.431** | **0.0187** |
| Easy High Reliability - Hard High Reliability | **1.434** | **0.1534** | **33** | **9.347** | **<.0001** |

**Table of Bonferroni Analyses – Automation x Explicability x Reliability**

| Conditions compared | Estimate | Standard Error | DF | t-ratio | P-value |
| --- | --- | --- | --- | --- | --- |
| Forced Unguided Medium Reliability - Free Unguided Medium Reliability | **-0.30957** | **0.0883** | **33** | **-3.507** | **0.0505** |
| Forced Unguided Medium Reliability - Forced Guided Medium Reliability | -0.03105 | 0.0673 | 33 | -0.462 | 1.0000 |
| Forced Unguided Medium Reliability - Free Guided Medium Reliability | **-0.51617** | **0.0975** | **33** | **-5.296** | **0.0004** |
| Forced Unguided Medium Reliability - Forced Unguided Low Reliability | **0.51829** | **0.0823** | **33** | **6.296** | **<.0001** |
| Forced Unguided Medium Reliability - Free Unguided Low Reliability | **-0.36072** | **0.0999** | **33** | **-3.611** | **0.0394** |
| Forced Unguided Medium Reliability - Forced Guided Low Reliability | 0.31910 | 0.1204 | 33 | 2.651 | 0.2936 |
| Forced Unguided Medium Reliability - Free Guided Low Reliability | **-0.43694** | **0.0963** | **33** | **-4.538** | **0.0035** |
| Forced Unguided Medium Reliability - Forced Unguided High Reliability | **-0.34791** | **0.0786** | **33** | **-4.424** | **0.0048** |
| Forced Unguided Medium Reliability - Free Unguided High Reliability | **-0.45544** | **0.1041** | **33** | **-4.375** | **0.0055** |
| Forced Unguided Medium Reliability - Forced Guided High Reliability | **-0.43122** | **0.1073** | **33** | **-4.018** | **0.0141** |
| Forced Unguided Medium Reliability - Free Guided High Reliability | **-0.67760** | **0.1045** | **33** | **-6.485** | **<.0001** |
| Free Unguided Medium Reliability - Forced Guided Medium Reliability | 0.27852 | 0.0968 | 33 | 2.877 | 0.1957 |
| Free Unguided Medium Reliability - Free Guided Medium Reliability | -0.20659 | 0.0813 | 33 | -2.541 | 0.3512 |
| Free Unguided Medium Reliability - Forced Unguided Low Reliability | **0.82786** | **0.1233** | **33** | **6.712** | **<.0001** |
| Free Unguided Medium Reliability - Free Unguided Low Reliability | -0.05115 | 0.0765 | 33 | -0.668 | 0.9999 |
| Free Unguided Medium Reliability - Forced Guided Low Reliability | **0.62867** | **0.1485** | **33** | **4.233** | **0.0080** |
| Free Unguided Medium Reliability - Free Guided Low Reliability | -0.12737 | 0.0771 | 33 | -1.651 | 0.8769 |
| Free Unguided Medium Reliability - Forced Unguided High Reliability | -0.03834 | 0.0867 | 33 | -0.442 | 1.0000 |
| Free Unguided Medium Reliability - Free Unguided High Reliability | -0.14586 | 0.0695 | 33 | -2.099 | 0.6268 |
| Free Unguided Medium Reliability - Forced Guided High Reliability | -0.12164 | 0.1139 | 33 | -1.068 | 0.9942 |
| Free Unguided Medium Reliability - Free Guided High Reliability | **-0.36802** | **0.0983** | **33** | **-3.745** | **0.0284** |
| Forced Guided Medium Reliability - Free Guided Medium Reliability | **-0.48511** | **0.0818** | **33** | **-5.934** | **0.0001** |
| Forced Guided Medium Reliability - Forced Unguided Low Reliability | **0.54934** | **0.0642** | **33** | **8.551** | **<.0001** |
| Forced Guided Medium Reliability - Free Unguided Low Reliability | **-0.32967** | **0.0927** | **33** | **-3.555** | **0.0451** |
| Forced Guided Medium Reliability - Forced Guided Low Reliability | 0.35015 | 0.1147 | 33 | 3.052 | 0.1384 |
| Forced Guided Medium Reliability - Free Guided Low Reliability | **-0.40589** | **0.0968** | **33** | **-4.194** | **0.0089** |
| Forced Guided Medium Reliability - Forced Unguided High Reliability | **-0.31686** | **0.0705** | **33** | **-4.492** | **0.0040** |
| Forced Guided Medium Reliability - Free Unguided High Reliability | **-0.42438** | **0.1122** | **33** | **-3.782** | **0.0259** |
| Forced Guided Medium Reliability - Forced Guided High Reliability | **-0.40016** | **0.0893** | **33** | **-4.481** | **0.0041** |
| Forced Guided Medium Reliability - Free Guided High Reliability | -0.64654 | 0.0944 | 33 | -6.846 | **<.0001** |
| Free Guided Medium Reliability - Forced Unguided Low Reliability | **1.03446** | **0.1098** | **33** | **9.419** | **<.0001** |
| Free Guided Medium Reliability - Free Unguided Low Reliability | 0.15544 | 0.0811 | 33 | 1.918 | 0.7405 |
| Free Guided Medium Reliability - Forced Guided Low Reliability | **0.83526** | **0.1477** | **33** | **5.655** | **0.0001** |
| Free Guided Medium Reliability - Free Guided Low Reliability | 0.07923 | 0.0862 | 33 | 0.920 | 0.9984 |
| Free Guided Medium Reliability - Forced Unguided High Reliability | 0.16825 | 0.0850 | 33 | 1.979 | 0.7033 |
| Free Guided Medium Reliability - Free Unguided High Reliability | 0.06073 | 0.0870 | 33 | 0.698 | 0.9999 |
| Free Guided Medium Reliability - Forced Guided High Reliability | 0.08495 | 0.0933 | 33 | 0.910 | 0.9985 |
| Free Guided Medium Reliability - Free Guided High Reliability | -0.16143 | 0.0774 | 33 | -2.085 | 0.6362 |
| Forced Unguided Low Reliability - Free Unguided Low Reliability | **-0.87901** | **0.1076** | **33** | **-8.171** | **<.0001** |
| Forced Unguided Low Reliability - Forced Guided Low Reliability | -0.19919 | 0.1076 | 33 | -1.852 | 0.7785 |
| Forced Unguided Low Reliability - Free Guided Low Reliability | **-0.95523** | **0.1150** | **33** | **-8.305** | **<.0001** |
| Forced Unguided Low Reliability - Forced Unguided High Reliability | **-0.86620** | **0.1017** | **33** | **-8.520** | **<.0001** |
| Forced Unguided Low Reliability - Free Unguided High Reliability | **-0.97373** | **0.1351** | **33** | **-7.210** | **<.0001** |
| Forced Unguided Low Reliability - Forced Guided High Reliability | **-0.94951** | **0.1156** | **33** | **-8.211** | **<.0001** |
| Forced Unguided Low Reliability - Free Guided High Reliability | **-1.19589** | **0.1282** | **33** | **-9.330** | **<.0001** |
| Free Unguided Low Reliability - Forced Guided Low Reliability | **0.67982** | **0.1621** | **33** | **4.194** | **0.0089** |
| Free Unguided Low Reliability - Free Guided Low Reliability | -0.07622 | 0.0958 | 33 | -0.796 | 0.9996 |
| Free Unguided Low Reliability - Forced Unguided High Reliability | 0.01281 | 0.0761 | 33 | 0.168 | 1.0000 |
| Free Unguided Low Reliability - Free Unguided High Reliability | -0.09472 | 0.0855 | 33 | -1.108 | 0.9921 |
| Free Unguided Low Reliability - Forced Guided High Reliability | -0.07049 | 0.0963 | 33 | -0.732 | 0.9998 |
| Free Unguided Low Reliability - Free Guided High Reliability | -0.31687 | 0.0917 | 33 | -3.456 | 0.0569 |
| Forced Guided Low Reliability - Free Guided Low Reliability | **-0.75604** | **0.1141** | **33** | **-6.627** | **<.0001** |
| Forced Guided Low Reliability - Forced Unguided High Reliability | **-0.66701** | **0.1443** | **33** | **-4.622** | **0.0028** |
| Forced Guided Low Reliability - Free Unguided High Reliability | **-0.77453** | **0.1574** | **33** | **-4.919** | **0.0012** |
| Forced Guided Low Reliability - Forced Guided High Reliability | **-0.75031** | **0.1641** | **33** | **-4.573** | **0.0032** |
| Forced Guided Low Reliability - Free Guided High Reliability | **-0.99669** | **0.1551** | **33** | **-6.428** | **<.0001** |
| Free Guided Low Reliability - Forced Unguided High Reliability | 0.08903 | 0.1049 | 33 | 0.848 | 0.9992 |
| Free Guided Low Reliability - Free Unguided High Reliability | -0.01850 | 0.0854 | 33 | -0.217 | 1.0000 |
| Free Guided Low Reliability - Forced Guided High Reliability | 0.00572 | 0.1163 | 33 | 0.049 | 1.0000 |
| Free Guided Low Reliability - Free Guided High Reliability | -0.24066 | 0.1000 | 33 | -2.406 | 0.4295 |
| Forced Unguided High Reliability - Free Unguided High Reliability | -0.10752 | 0.0899 | 33 | -1.195 | 0.9857 |
| Forced Unguided High Reliability - Forced Guided High Reliability | -0.08330 | 0.0961 | 33 | -0.867 | 0.9990 |
| Forced Unguided High Reliability - Free Guided High Reliability | **-0.32968** | **0.0915** | **33** | **-3.603** | **0.0401** |
| Free Unguided High Reliability - Forced Guided High Reliability | **0.02422** | **0.1230** | **33** | **0.197** | **1.0000** |
| Free Unguided High Reliability - Free Guided High Reliability | -0.22216 | 0.1062 | 33 | -2.092 | 0.6316 |
| Forced Guided High Reliability - Free Guided High Reliability | **-0.24638** | **0.0627** | **33** | **-3.931** | **0.0177** |
| Forced Easy Medium Reliability - Free Easy Medium Reliability | **-0.3501** | 0.0846 | 33 | -4.136 | **0.0104** |
| Forced Easy Medium Reliability - Forced Hard Medium Reliability | **1.2664** | **0.1404** | **33** | **9.017** | **0.0001** |
| Forced Easy Medium Reliability - Free Hard Medium Reliability | **0.8218** | 0.1484 | 33 | 5.536 | **0.0002** |
| Forced Easy Medium Reliability - Forced Easy Low Reliability | **0.6799** | 0.0880 | 33 | 7.723 | **0.0001** |
| Forced Easy Medium Reliability - Free Easy Low Reliability | **-0.3803** | 0.0828 | 33 | -4.594 | **0.0030** |
| Forced Easy Medium Reliability - Forced Hard Low Reliability | **1.4549** | 0.1428 | 33 | 10.187 | **0.0001** |
| Forced Easy Medium Reliability - Free Hard Low Reliability | **0.8801** | 0.1753 | 33 | 5.022 | **0.0009** |
| Forced Easy Medium Reliability - Forced Easy High Reliability | **-0.4809** | 0.0887 | 33 | -5.422 | **0.0003** |
| Forced Easy Medium Reliability - Free Easy High Reliability | **-0.6119** | 0.0842 | 33 | -7.264 | **0.0001** |
| Forced Easy Medium Reliability - Forced Hard High Reliability | **0.9992** | **0.1601** | **33** | **6.240** | **0.0001** |
| Forced Easy Medium Reliability - Free Hard High Reliability | **0.7764** | 0.1773 | 33 | 4.379 | **0.0054** |
| Free Easy Medium Reliability - Forced Hard Medium Reliability | **1.6165** | 0.1455 | 33 | 11.108 | **0.0001** |
| Free Easy Medium Reliability - Free Hard Medium Reliability | **1.1720** | 0.1356 | 33 | 8.641 | **0.0001** |
| Free Easy Medium Reliability - Forced Easy Low Reliability | **1.0300** | 0.1351 | 33 | 7.624 | **0.0001** |
| Free Easy Medium Reliability - Free Easy Low Reliability | -0.0302 | 0.0487 | 33 | -0.621 | 1.0000 |
| Free Easy Medium Reliability - Forced Hard Low Reliability | **1.8051** | 0.1597 | 33 | 11.304 | **0.0001** |
| Free Easy Medium Reliability - Free Hard Low Reliability | **1.2303** | 0.1633 | 33 | 7.533 | **0.0001** |
| Free Easy Medium Reliability - Forced Easy High Reliability | **-0.1307** | 0.0794 | 33 | -1.646 | **0.8789** |
| Free Easy Medium Reliability - Free Easy High Reliability | **-0.2618** | 0.0582 | 33 | -4.499 | **0.0039** |
| Free Easy Medium Reliability - Forced Hard High Reliability | **1.3493** | 0.1532 | 33 | 8.805 | **0.0001** |
| Free Easy Medium Reliability - Free Hard High Reliability | **1.1265** | 0.1486 | 33 | 7.579 | **0.0001** |
| Forced Hard Medium Reliability - Free Hard Medium Reliability | **-0.4446** | **0.0998** | **33** | **-4.454** | **0.0044** |
| Forced Hard Medium Reliability - Forced Easy Low Reliability | **-0.5865** | **0.1656** | **33** | **-3.543** | **0.0464** |
| Forced Hard Medium Reliability - Free Easy Low Reliability | **-1.6467** | 0.1438 | 33 | -11.449 | **0.0001** |
| Forced Hard Medium Reliability - Forced Hard Low Reliability | 0.1886 | 0.0815 | 33 | 2.314 | 0.4870 |
| Forced Hard Medium Reliability - Free Hard Low Reliability | **-0.3863** | 0.0985 | 33 | -3.922 | **0.0181** |
| Forced Hard Medium Reliability - Forced Easy High Reliability | **-1.7473** | 0.1490 | 33 | -11.725 | **0.0001** |
| Forced Hard Medium Reliability - Free Easy High Reliability | **-1.8783** | 0.1569 | 33 | -11.974 | **0.0001** |
| Forced Hard Medium Reliability - Forced Hard High Reliability | **-0.2672** | **0.0716** | **33** | **-3.734** | **0.0291** |
| Forced Hard Medium Reliability - Free Hard High Reliability | **-0.4900** | 0.1090 | 33 | -4.494 | **0.0040** |
| Free Hard Medium Reliability - Forced Easy Low Reliability | **-0.1419** | 0.1740 | 33 | -0.816 | **0.9995** |
| Free Hard Medium Reliability - Free Easy Low Reliability | **-1.2022** | 0.1308 | 33 | -9.193 | **0.0001** |
| Free Hard Medium Reliability - Forced Hard Low Reliability | **0.6331** | 0.1316 | 33 | 4.810 | **0.0017** |
| Free Hard Medium Reliability - Free Hard Low Reliability | 0.0583 | 0.0836 | 33 | 0.697 | 0.9999 |
| Free Hard Medium Reliability - Forced Easy High Reliability | **-1.3027** | 0.1569 | 33 | -8.300 | **0.0001** |
| Free Hard Medium Reliability - Free Easy High Reliability | **-1.4338** | 0.1550 | 33 | -9.253 | **0.0001** |
| Forced Hard Medium Reliability - Forced Hard High Reliability | **-0.2672** | **0.0716** | **33** | **-3.734** | **0.0291** |
| Forced Hard Medium Reliability - Free Hard High Reliability | **-0.4900** | 0.1090 | 33 | -4.494 | **0.0040** |
| Free Hard Medium Reliability - Forced Easy Low Reliability | **-0.1419** | 0.1740 | 33 | -0.816 | **0.9995** |
| Free Hard Medium Reliability - Free Easy Low Reliability | **-1.2022** | 0.1308 | 33 | -9.193 | **0.0001** |
| Free Hard Medium Reliability - Forced Hard Low Reliability | **0.6331** | 0.1316 | 33 | 4.810 | **0.0017** |
| Free Hard Medium Reliability - Free Hard Low Reliability | 0.0583 | 0.0836 | 33 | 0.697 | 0.9999 |
| Free Hard Medium Reliability - Forced Easy High Reliability | **-1.3027** | 0.1569 | 33 | -8.300 | **0.0001** |
| Free Hard Medium Reliability - Free Easy High Reliability | **-1.4338** | 0.1550 | 33 | -9.253 | **0.0001** |
| Forced Hard High Reliability - Free Hard High Reliability | -0.2228 | 0.0893 | 33 | -2.495 | 0.3773 |

**Table of Bonferroni Analyses – Automation x Difficulty x Reliability x Explicability**

| Conditions compared | estimate | Standard Error | DF | t.ratio | P.value |
| --- | --- | --- | --- | --- | --- |
| Forced Easy Medium Reliability Unguided - Free Easy Medium Reliability Unguided | **-0.34714** | 0.0829 | 33 | -4.186 | **0.0285** |
| Forced Easy Medium Reliability Unguided - Forced Hard Medium Reliability Unguided | **1.13524** | 0.1487 | 33 | 7.635 | **<.0001** |
| Forced Easy Medium Reliability Unguided - Free Hard Medium Reliability Unguided | **0.86324** | 0.1709 | 33 | 5.052 | **0.0029** |
| Forced Easy Medium Reliability Unguided - Forced Easy Low Reliability Unguided | **0.70773** | 0.1047 | 33 | 6.759 | **<.0001** |
| Forced Easy Medium Reliability Unguided - Free Easy Low Reliability Unguided | **-0.40635** | 0.0924 | 33 | -4.398 | **0.0166** |
| Forced Easy Medium Reliability Unguided - Forced Hard Low Reliability Unguided | **1.46409** | 0.1520 | 33 | 9.629 | **<.0001** |
| Forced Easy Medium Reliability Unguided - Free Hard Low Reliability Unguided | **0.82015** | 0.1777 | 33 | 4.615 | **0.0094** |
| Forced Easy Medium Reliability Unguided - Forced Easy High Reliability Unguided | **-0.53226** | 0.0975 | 33 | -5.460 | **0.0009** |
| Forced Easy Medium Reliability Unguided - Free Easy High Reliability Unguided | **-0.49552** | 0.0946 | 33 | -5.239 | **0.0017** |
| Forced Easy Medium Reliability Unguided - Forced Hard High Reliability Unguided | **0.97167** | 0.1476 | 33 | 6.583 | **<.0001** |
| Forced Easy Medium Reliability Unguided - Free Hard High Reliability Unguided | **0.71989** | 0.1748 | 33 | 4.119 | **0.0336** |
| Forced Easy Medium Reliability Unguided - Forced Easy Medium Reliability Guided | -0.16220 | 0.0681 | 33 | -2.383 | 0.7428 |
| Forced Easy Medium Reliability Unguided - Free Easy Medium Reliability Guided | **-0.51532** | 0.0989 | 33 | -5.208 | **0.0019** |
| Forced Easy Medium Reliability Unguided - Forced Hard Medium Reliability Guided | **1.23534** | 0.1452 | 33 | 8.506 | **<.0001** |
| Forced Easy Medium Reliability Unguided - Free Hard Medium Reliability Guided | **0.68462** | 0.1613 | 33 | 4.243 | **0.0121** |
| Forced Easy Medium Reliability Unguided - Forced Easy Low Reliability Guided | **0.81111** | 0.1005 | 33 | 8.076 | **<.0001** |
| Forced Easy Medium Reliability Unguided - Free Easy Low Reliability Guided | **-0.37728** | 0.0861 | 33 | -4.379 | **0.0162** |
| Forced Easy Medium Reliability Unguided - Forced Hard Low Reliability Guided | **1.56890** | 0.1441 | 33 | 10.878 | **<.0001** |
| Forced Easy Medium Reliability Unguided - Free Hard Low Reliability Guided | **0.76517** | 0.1653 | 33 | 4.630 | **0.0091** |
| Forced Easy Medium Reliability Unguided - Forced Easy High Reliability Guided | **-0.43029** | 0.0905 | 33 | -4.749 | **0.0080** |
| Forced Easy Medium Reliability Unguided - Free Easy High Reliability Guided | **-0.42165** | 0.0893 | 33 | -4.717 | **0.0084** |
| Forced Easy Medium Reliability Unguided - Forced Hard High Reliability Guided | **1.10758** | 0.1440 | 33 | 7.690 | **<.0001** |
| Forced Easy Medium Reliability Unguided - Free Hard High Reliability Guided | **0.64824** | 0.1681 | 33 | 3.858 | **0.0499** |
| Forced Hard Medium Reliability Unguided - Free Hard Medium Reliability Unguided | -0.27200 | 0.1139 | 33 | -2.386 | 0.7417 |
| Forced Hard Medium Reliability Unguided - Forced Easy Low Reliability Unguided | **-0.42751** | 0.0802 | 33 | -5.330 | **0.0011** |
| Forced Hard Medium Reliability Unguided - Free Easy Low Reliability Unguided | **-1.54159** | 0.1369 | 33 | -11.25 | **<.0001** |
| Forced Hard Medium Reliability Unguided - Forced Hard Low Reliability Unguided | 0.32886 | 0.1260 | 33 | 2.610 | 0.5959 |
| Forced Hard Medium Reliability Unguided - Free Hard Low Reliability Unguided | **-0.31509** | 0.1426 | 33 | -2.209 | 0.9941 |
| Forced Hard Medium Reliability Unguided - Forced Easy High Reliability Unguided | **-0.37980** | **0.0861** | **33** | **-4.409** | **0.0156** |
| Forced Hard Medium Reliability Unguided - Free Easy High Reliability Unguided | **-0.34306** | 0.0834 | 33 | -4.114 | **0.0334** |
| Forced Hard Medium Reliability Unguided - Forced Hard High Reliability Unguided | 0.17492 | 0.1249 | 33 | 1.400 | 0.9829 |
| Forced Hard Medium Reliability Unguided - Free Hard High Reliability Unguided | -0.08735 | 0.1411 | 33 | -0.619 | 0.9898 |
| Free Easy Medium Reliability Unguided - Forced Easy Low Reliability Unguided | **-0.15663** | 0.0776 | 33 | -2.019 | 0.8807 |
| Free Easy Medium Reliability Unguided - Free Easy Low Reliability Unguided | **-0.90189** | 0.1306 | 33 | -6.903 | **<.0001** |
| Free Easy Medium Reliability Unguided - Forced Hard Low Reliability Unguided | 0.05526 | 0.1200 | 33 | 0.461 | 0.9867 |
| Free Easy Medium Reliability Unguided - Free Hard Low Reliability Unguided | -0.18791 | 0.1360 | 33 | -1.381 | 0.9860 |
| Free Easy Medium Reliability Unguided - Forced Easy High Reliability Unguided | -0.08225 | 0.0719 | 33 | -1.141 | 0.9986 |
| Free Easy Medium Reliability Unguided - Free Easy High Reliability Unguided | -0.04551 | 0.0698 | 33 | -0.653 | 0.9866 |
| Free Easy Medium Reliability Unguided - Forced Hard High Reliability Unguided | 0.26524 | 0.1186 | 33 | 2.238 | 0.5537 |
| Free Easy Medium Reliability Unguided - Free Hard High Reliability Unguided | 0.01746 | 0.1323 | 33 | 0.132 | 0.9909 |
| Forced Easy Low Reliability Unguided - Free Easy Low Reliability Unguided | **-0.74526** | **0.1053** | **33** | **-7.078** | **<.0001** |
| Forced Easy Low Reliability Unguided - Forced Hard Low Reliability Unguided | 0.21152 | 0.0937 | 33 | 2.255 | 0.5489 |
| Forced Easy Low Reliability Unguided - Free Hard Low Reliability Unguided | **-0.47025** | **0.1264** | **33** | **-3.720** | **0.0481** |
| Forced Easy Low Reliability Unguided - Forced Easy High Reliability Unguided | 0.07437 | 0.0816 | 33 | 0.911 | 0.9999 |
| Forced Easy Low Reliability Unguided - Free Easy High Reliability Unguided | 0.11111 | 0.0797 | 33 | 1.394 | 0.9828 |
| Forced Easy Low Reliability Unguided - Forced Hard High Reliability Unguided | **0.42176** | 0.0937 | 33 | 4.499 | **0.0077** |
| Forced Easy Low Reliability Unguided - Free Hard High Reliability Unguided | 0.16958 | 0.1192 | 33 | 1.422 | 0.9775 |
| Free Easy Low Reliability Unguided - Forced Hard Low Reliability Unguided | **0.95678** | 0.1086 | 33 | 8.817 | **<.0001** |
| Free Easy Low Reliability Unguided - Free Hard Low Reliability Unguided | 0.27501 | 0.1343 | 33 | 2.048 | 0.8663 |
| Free Easy Low Reliability Unguided - Forced Easy High Reliability Unguided | 0.15689 | 0.0878 | 33 | 1.786 | 0.9577 |
| Free Easy Low Reliability Unguided - Free Easy High Reliability Unguided | 0.12015 | 0.0859 | 33 | 1.399 | 0.9830 |
| Free Easy Low Reliability Unguided - Forced Hard High Reliability Unguided | **0.54653** | 0.1086 | 33 | 5.035 | **0.0032** |
| Free Easy Low Reliability Unguided - Free Hard High Reliability Unguided | 0.09435 | 0.1244 | 33 | 0.758 | 0.9981 |
| Forced Hard Low Reliability Unguided - Free Hard Low Reliability Unguided | **-0.68177** | 0.1202 | 33 | -5.671 | **0.0003** |
| Forced Hard Low Reliability Unguided - Forced Easy High Reliability Unguided | 0.08265 | 0.0905 | 33 | 0.913 | 0.9999 |
| Forced Hard Low Reliability Unguided - Free Easy High Reliability Unguided | 0.04591 | 0.0883 | 33 | 0.520 | 0.9879 |
| Forced Hard Low Reliability Unguided - Forced Hard High Reliability Unguided | 0.32229 | 0.1202 | 33 | 2.681 | 0.4657 |
| Forced Hard Low Reliability Unguided - Free Hard High Reliability Unguided | -0.04441 | 0.1345 | 33 | -0.330 | 0.9933 |
| Free Hard Low Reliability Unguided - Forced Easy High Reliability Unguided | -0.04074 | 0.0721 | 33 | -0.565 | 0.9927 |
| Free Hard Low Reliability Unguided - Free Easy High Reliability Unguided | -0.07748 | 0.0701 | 33 | -1.106 | 0.9985 |
| Free Hard Low Reliability Unguided - Forced Hard High Reliability Unguided | 0.16670 | 0.1174 | 33 | 1.419 | 0.9780 |
| Free Hard Low Reliability Unguided - Free Hard High Reliability Unguided | -0.08794 | 0.1311 | 33 | -0.671 | 0.9967 |
| Forced Easy High Reliability Unguided - Free Easy High Reliability Unguided | -0.03674 | 0.0742 | 33 | -0.495 | 0.9946 |
| Forced Easy High Reliability Unguided - Forced Hard High Reliability Unguided | 0.23904 | 0.1014 | 33 | 2.358 | 0.5779 |
| Forced Easy High Reliability Unguided - Free Hard High Reliability Unguided | -0.00184 | 0.1166 | 33 | -0.016 | 1.0000 |
| Free Easy High Reliability Unguided - Forced Hard High Reliability Unguided | 0.27579 | 0.0999 | 33 | 2.759 | 0.3678 |
| Free Easy High Reliability Unguided - Free Hard High Reliability Unguided | 0.03490 | 0.1142 | 33 | 0.306 | 0.9972 |
| Forced Hard High Reliability Unguided - Free Hard High Reliability Unguided | -0.24093 | 0.1236 | 33 | -1.949 | 0.8507 |

### Agreement Score

**Table of Bonferroni Analyses – Reliability**

| Conditions compared | estimate | Standard Error | DF | t.ratio | P.value |
| --- | --- | --- | --- | --- | --- |
| Medium Reliability - Low Reliability | **0.343** | **0.0368** | **33** | **9.315** | **<.0001** |
| Medium Reliability - High Reliability | **-0.323** | **0.0378** | **33** | **-8.562** | **<.0001** |
| Low Reliability - High Reliability | **-0.666** | **0.0566** | **33** | **-11.764** | **<.0001** |

**Table of Bonferroni Analyses – Explicability x Reliability**

| Conditions compared | estimate | Standard Error | DF | t.ratio | P.value |
| --- | --- | --- | --- | --- | --- |
| Unguided Medium Reliability - Guided Medium Reliability | 0.0154 | 0.0468 | 33 | 0.330 | 0.9994 |
| Unguided Medium Reliability - Unguided Low Reliability | **0.3039** | **0.0460** | **33** | **6.613** | **<.0001** |
| Unguided Medium Reliability - Guided Low Reliability | **0.3969** | **0.0618** | **33** | **6.422** | **<.0001** |
| Unguided Medium Reliability - Unguided High Reliability | **-0.2427** | **0.0375** | **33** | **-6.465** | **<.0001** |
| Unguided Medium Reliability - Guided High Reliability | **-0.3886** | **0.0654** | **33** | **-5.946** | **<.0001** |
| Guided Medium Reliability - Unguided Low Reliability | **0.2885** | **0.0350** | **33** | **8.239** | **<.0001** |
| Guided Medium Reliability - Guided Low Reliability | **0.3815** | **0.0563** | **33** | **6.773** | **<.0001** |
| Guided Medium Reliability - Unguided High Reliability | **-0.2581** | **0.0470** | **33** | **-5.488** | **0.0001** |
| Guided Medium Reliability - Guided High Reliability | **-0.4040** | **0.0526** | **33** | **-7.676** | **<.0001** |
| Unguided Low Reliability - Guided Low Reliability | 0.0930 | 0.0522 | 33 | 1.781 | 0.4910 |
| Unguided Low Reliability - Unguided High Reliability | **-0.5466** | **0.0523** | **33** | **-10.447** | **<.0001** |
| Unguided Low Reliability - Guided High Reliability | **-0.6925** | **0.0583** | **33** | **-11.887** | **<.0001** |
| Guided Low Reliability - Unguided High Reliability | **-0.6396** | **0.0675** | **33** | **-9.480** | **<.0001** |
| Guided Low Reliability - Guided High Reliability | **-0.7855** | **0.0874** | **33** | **-8.989** | **<.0001** |
| Unguided High Reliability - Guided High Reliability | -0.1459 | 0.0526 | 33 | -2.772 | 0.0877 |

**Table of Bonferroni Analyses – Explicability x Difficulty x Reliability**

| Conditions compared | Estimate | Standard Error | DF | t.ratio | P.value |
| --- | --- | --- | --- | --- | --- |
| Unguided Easy Medium Reliability - Guided Easy Medium Reliability | -0.03822 | 0.0537 | 33 | -0.712 | 0.9998 |
| Unguided Easy Medium Reliability - Unguided Hard Medium Reliability | **0.39687** | **0.0726** | **33** | **5.469** | **0.0003** |
| Unguided Easy Medium Reliability - Guided Hard Medium Reliability | **0.46596** | **0.0820** | **33** | **5.684** | **0.0001** |
| Unguided Easy Medium Reliability - Unguided Easy Low Reliability | **0.42328** | **0.0572** | **33** | **7.396** | **<.0001** |
| Unguided Easy Medium Reliability - Guided Easy Low Reliability | **0.45632** | **0.0735** | **33** | **6.210** | **<.0001** |
| Unguided Easy Medium Reliability - Unguided Hard Low Reliability | **0.58139** | **0.0784** | **33** | **7.420** | **<.0001** |
| Unguided Easy Medium Reliability - Guided Hard Low Reliability | **0.73439** | **0.0825** | **33** | **8.901** | **<.0001** |
| Unguided Easy Medium Reliability - Unguided Easy High Reliability | **-0.40936** | **0.0546** | **33** | **-7.491** | **<.0001** |
| Unguided Easy Medium Reliability - Guided Easy High Reliability | **-0.50416** | **0.0794** | **33** | **-6.346** | **<.0001** |
| Unguided Easy Medium Reliability - Unguided Hard High Reliability | **0.32084** | **0.0615** | **33** | **5.220** | **0.0005** |
| Unguided Easy Medium Reliability - Guided Hard High Reliability | 0.12384 | 0.0748 | 33 | 1.656 | 0.8747 |
| Guided Easy Medium Reliability - Unguided Hard Medium Reliability | **0.43509** | **0.0741** | **33** | **5.869** | **0.0001** |
| Guided Easy Medium Reliability - Guided Hard Medium Reliability | **0.50417** | **0.0689** | **33** | **7.319** | **<.0001** |
| Guided Easy Medium Reliability - Unguided Easy Low Reliability | **0.46150** | **0.0420** | **33** | **10.977** | **<.0001** |
| Guided Easy Medium Reliability - Guided Easy Low Reliability | **0.49453** | **0.0705** | **33** | **7.017** | **<.0001** |
| Guided Easy Medium Reliability - Unguided Hard Low Reliability | **0.61960** | **0.0668** | **33** | **9.270** | **<.0001** |
| Guided Easy Medium Reliability - Guided Hard Low Reliability | **0.77260** | **0.0806** | **33** | **9.582** | **<.0001** |
| Guided Easy Medium Reliability - Unguided Easy High Reliability | **-0.37114** | **0.0576** | **33** | **-6.444** | **<.0001** |
| Guided Easy Medium Reliability - Guided Easy High Reliability | **-0.46595** | **0.0657** | **33** | **-7.091** | **<.0001** |
| Guided Easy Medium Reliability - Unguided Hard High Reliability | **0.35906** | **0.0688** | **33** | **5.222** | **0.0005** |
| Guided Easy Medium Reliability - Guided Hard High Reliability | 0.16206 | 0.0646 | 33 | 2.507 | 0.3702 |
| Unguided Hard Medium Reliability - Guided Hard Medium Reliability | 0.06909 | 0.0606 | 33 | 1.141 | 0.9901 |
| Unguided Hard Medium Reliability - Unguided Easy Low Reliability | 0.02641 | 0.0628 | 33 | 0.420 | 1.0000 |
| Unguided Hard Medium Reliability - Guided Easy Low Reliability | 0.05945 | 0.0962 | 33 | 0.618 | 1.0000 |
| Unguided Hard Medium Reliability - Unguided Hard Low Reliability | 0.18452 | 0.0581 | 33 | 3.174 | 0.1071 |
| Unguided Hard Medium Reliability - Guided Hard Low Reliability | **0.33752** | **0.0722** | **33** | **4.676** | **0.0024** |
| Unguided Hard Medium Reliability - Unguided Easy High Reliability | **-0.80622** | **0.0823** | **33** | **-9.797** | **<.0001** |
| Unguided Hard Medium Reliability - Guided Easy High Reliability | **-0.90103** | **0.0973** | **33** | **-9.264** | **<.0001** |
| Unguided Hard Medium Reliability - Unguided Hard High Reliability | -0.07603 | 0.0590 | 33 | -1.288 | 0.9750 |
| Unguided Hard Medium Reliability - Guided Hard High Reliability | -0.27303 | 0.0807 | 33 | -3.383 | 0.0673 |
| Guided Hard Medium Reliability - Unguided Easy Low Reliability | -0.04268 | 0.0610 | 33 | -0.700 | 0.9999 |
| Guided Hard Medium Reliability - Guided Easy Low Reliability | -0.00964 | 0.0862 | 33 | -0.112 | 1.0000 |
| Unguided Hard Medium Reliability - Unguided Hard Low Reliability | 0.11543 | 0.0522 | 33 | 2.211 | 0.5540 |
| Unguided Hard Medium Reliability - Guided Hard Low Reliability | **0.26843** | **0.0683** | **33** | **3.931** | **0.0177** |
| Unguided Hard Medium Reliability - Unguided Easy High Reliability | **-0.87531** | **0.0873** | **33** | **-10.024** | **<.0001** |
| Unguided Hard Medium Reliability - Guided Easy High Reliability | **-0.97012** | **0.0907** | **33** | **-10.692** | **<.0001** |
| Unguided Hard Medium Reliability - Unguided Hard High Reliability | -0.14512 | 0.0636 | 33 | -2.283 | 0.5070 |
| Unguided Hard Medium Reliability - Guided Hard High Reliability | **-0.34212** | **0.0680** | **33** | **-5.034** | **0.0009** |
| Guided Hard Medium Reliability - Unguided Easy High Reliability | **-1.14374** | **0.1033** | **33** | **-11.076** | **<.0001** |
| Guided Hard Medium Reliability - Guided Easy High Reliability | **-1.23855** | **0.1135** | **33** | **-10.913** | **<.0001** |
| Guided Hard Medium Reliability - Unguided Hard High Reliability | -0.14512 | 0.0636 | 33 | -2.283 | 0.5070 |
| Guided Hard Medium Reliability - Guided Hard High Reliability | **-0.34212** | **0.0680** | **33** | **-5.034** | **0.0009** |
| Unguided Easy Low Reliability - Guided Easy Low Reliability | 0.03304 | 0.0664 | 33 | 0.498 | 1.0000 |
| Unguided Easy Low Reliability - Unguided Hard Low Reliability | 0.15811 | 0.0550 | 33 | 2.873 | 0.1971 |
| Unguided Easy Low Reliability - Guided Hard Low Reliability | **0.31111** | **0.0601** | **33** | **5.172** | **0.0006** |
| Unguided Easy Low Reliability - Unguided Easy High Reliability | **-0.83263** | **0.0626** | **33** | **-13.292** | **<.0001** |
| Unguided Easy Low Reliability - Guided Easy High Reliability | **-0.92744** | **0.0725** | **33** | **-12.785** | **<.0001** |
| Unguided Easy Low Reliability - Unguided Hard High Reliability | -0.10244 | 0.0619 | 33 | -1.654 | 0.8756 |
| Unguided Easy Low Reliability - Guided Hard High Reliability | **-0.29944** | **0.0637** | **33** | **-4.704** | **0.0022** |
| Guided Easy Low Reliability - Unguided Hard Low Reliability | 0.12507 | 0.0887 | 33 | 1.410 | 0.9532 |
| Guided Easy Low Reliability - Guided Hard Low Reliability | **0.27807** | **0.0783** | **33** | **3.553** | **0.0453** |
| Guided Easy Low Reliability - Unguided Easy High Reliability | **-0.86567** | **0.0867** | **33** | **-9.989** | **<.0001** |
| Guided Easy Low Reliability - Guided Easy High Reliability | **-0.96048** | **0.1026** | **33** | **-9.364** | **<.0001** |
| Guided Easy Low Reliability - Unguided Hard High Reliability | -0.13548 | 0.0773 | 33 | -1.754 | 0.8303 |
| Guided Easy Low Reliability - Guided Hard High Reliability | -0.33247 | 0.0965 | 33 | -3.447 | 0.0581 |
| Unguided Hard Low Reliability - Guided Hard Low Reliability | 0.15300 | 0.0646 | 33 | 2.369 | 0.4527 |
| Unguided Hard Low Reliability - Unguided Easy High Reliability | **-0.99074** | **0.0918** | **33** | **-10.791** | **<.0001** |
| Unguided Hard Low Reliability - Guided Easy High Reliability | **-1.08555** | **0.0867** | **33** | **-12.522** | **<.0001** |
| Unguided Hard Low Reliability - Unguided Hard High Reliability | **-0.26055** | **0.0625** | **33** | **-4.170** | **0.0095** |
| Unguided Hard Low Reliability - Guided Hard High Reliability | **-0.45755** | **0.0728** | **33** | **-6.289** | **<.0001** |
| Guided Hard Low Reliability - Unguided Easy High Reliability | **-1.14374** | **0.1033** | **33** | **-11.076** | **<.0001** |
| Guided Hard Low Reliability - Guided Easy High Reliability | **-1.23855** | **0.1135** | **33** | **-10.913** | **<.0001** |
| Guided Hard Low Reliability - Unguided Hard High Reliability | **-0.41355** | **0.0794** | **33** | **-5.208** | **0.0005** |
| Guided Hard Low Reliability - Guided Hard High Reliability | **-0.61055** | **0.0974** | **33** | **-6.267** | **<.0001** |
| Unguided Easy High Reliability - Guided Easy High Reliability | -0.09481 | 0.0690 | 33 | -1.373 | 0.9608 |
| Unguided Easy High Reliability - Unguided Hard High Reliability | **0.73020** | **0.0782** | **33** | **9.338** | **<.0001** |
| Unguided Easy High Reliability - Guided Hard High Reliability | **0.53320** | **0.0745** | **33** | **7.153** | **<.0001** |
| Guided Easy High Reliability - Unguided Hard High Reliability | **0.82500** | **0.0850** | **33** | **9.705** | **<.0001** |
| Guided Easy High Reliability - Guided Hard High Reliability | **0.62800** | **0.0743** | **33** | **8.452** | **<.0001** |
| Unguided Hard High Reliability - Guided Hard High Reliability | -0.19700 | 0.0719 | 33 | -2.740 | 0.251 |

### Acceptability – Usefulness

**Table of Bonferroni Analyses – Reliability**

| Conditions compared | estimate | Standard Error | DF | t.ratio | P.value |
| --- | --- | --- | --- | --- | --- |
| Medium Reliability - Low Reliability | **10.3** | **1.87** | **33** | **5.527** | **<.0001** |
| Medium Reliability - High Fiable | **-11.3** | **1.70** | **33** | **-6.602** | **<.0001** |
| Low Reliability - High Fiable | **-21.6** | **2.39** | **33** | **-9.025** | **<.0001** |

### Acceptability – Satisfaction

**Table of Bonferroni Analyses – Reliability**

| Conditions compared | estimate | Standard Error | DF | t.ratio | P.value |
| --- | --- | --- | --- | --- | --- |
| Medium Reliability - Low Reliability | **20.9** | **1.95** | **33** | **10.752** | **<.0001** |
| Medium Reliability - High Fiable | **-14.5** | **1.68** | **33** | **-8.609** | **<.0001** |
| Low Reliability - High Fiable | **-35.4** | **2.05** | **33** | **-17.243** | **<.0001** |

### Reaction time

**Table of Bonferroni Analyses - Reliability**

| Condition tested | Estimate | Standard Error | DF | t-ratio | P-value |
| --- | --- | --- | --- | --- | --- |
| Medium Reliability Low Reliability | 1.37 | 13.6 | 33 | 0.101 | 0.9944 |
| Medium Reliability High Reliability | **150.44** | **16.7** | **33** | **8.995** | **<.0001** |
| Low ReliabilityHigh Reliability | **149.07** | **20.6** | **33** | **7.235** | **<.0001** |

**Table of Bonferroni Analyses – Difficulty x Explicability**

| Condition tested | Estimate | Standard Error | DF | t-ratio | P-value |
| --- | --- | --- | --- | --- | --- |
| Easy UnGuided x Hard UnGuided | **-220** | **41.4** | **33** | **-5.317** | **<.0001** |
| Easy UnGuided x Easy Guided | **-100** | **23.5** | **33** | **-4.273** | **0.0008** |
| Easy UnGuided x Hard Guided | **-395** | **46.8** | **33** | **-8.447** | **<.0001** |
| Hard UnGuided x Easy Guided | 120 | 41.6 | 33 | 2.875 | 0.0338 |
| Hard UnGuided x Hard Guided | **-175** | **28.8** | **33** | **-6.076** | **<.0001** |
| Easy Guided x Hard Guided | **-295** | **36.9** | **33** | **-7.987** | **<.0001** |

**Table of Bonferroni Analyses – Difficulty x Reliability**

| Condition tested | Estimate | Standard Error | DF | t-ratio | P-value |
| --- | --- | --- | --- | --- | --- |
| Easy Medium Reliability x Hard Medium Reliability | -267.6 | 42.2 | 33 | -6.337 | <.0001 |
| Easy Medium Reliability x Easy Low Reliability | -25.5 | 20.7 | 33 | -1.231 | 0.8185 |
| Easy Medium Reliability x Hard Low Reliability | -239.4 | 35.8 | 33 | -6.687 | <.0001 |
| Easy Medium Reliability x Easy High Reliability | 161.8 | 22.6 | 33 | 7.154 | <.0001 |
| Easy Medium Reliability x Hard High Reliability | -128.5 | 39.2 | 33 | -3.276 | 0.0274 |
| Hard Medium Reliability x Easy Low Reliability | 242.1 | 43.0 | 33 | 5.626 | <.0001 |
| Hard Medium Reliability x Hard Low Reliability | 28.2 | 20.4 | 33 | 1.383 | 0.7367 |
| Hard Medium Reliability x Easy High Reliability | **429.4** | **46.2** | **33** | **9.284** | **<.0001** |
| Hard Medium Reliability x Hard High Reliability | **139.1** | **20.2** | **33** | **6.882** | **<.0001** |
| Easy Low Reliability x Hard Low Reliability | **-213.9** | **38.1** | **33** | **-5.609** | **<.0001** |
| Easy Low Reliability x Easy High Reliability | **187.3** | **23.5** | **33** | **7.978** | **<.0001** |
| Easy Low Reliability x Hard High Reliability | -103.0 | 41.1 | 33 | -2.506 | 0.1517 |
| Hard Low Reliability x Easy High Reliability | **401.1** | **45.3** | **33** | **8.860** | **<.0001** |
| Hard Low Reliability x Hard High Reliability | **110.9** | **23.6** | **33** | **4.701** | **0.0006** |
| Easy High Reliability x Hard High Reliability | **-290.3** | **41.2** | **33** | **-7.051** | **<.0001** |

**Table of Bonferroni Analyses – Difficulty x Automation**

| Condition tested | Estimate | Standard Error | DF | t-ratio | P-value |
| --- | --- | --- | --- | --- | --- |
| Easy Forced x Hard Forced | **-197.5** | **40.0** | **33** | **-4.936** | **0.0001** |
| Easy Forced x Easy Free | **88.3** | **22.4** | **33** | **3.939** | **0.0022** |
| Easy Forced x Hard Free | **-228.7** | **44.6** | **33** | **-5.123** | **0.0001** |
| Hard Forced x Easy Free | **285.8** | **50.4** | **33** | **5.671** | **<.0001** |
| Hard Forced x Hard Free | -31.1 | 44.3 | 33 | -0.703 | 0.8952 |
| Easy Free x Hard Free | **-317.0** | **44.0** | **33** | **-7.206** | **<.0001** |

**Table of Bonferroni Analyses –Automation x Reliability**

| Condition tested | Estimate | Standard Error | DF | t-ratio | P-value |
| --- | --- | --- | --- | --- | --- |
| Forced Medium Reliability x Free Medium Reliability | 51.61 | 32.8 | 33 | 1.572 | 0.6221 |
| Forced Medium Reliability x Forced Low Reliability | -9.89 | 17.5 | 33 | -0.565 | 0.9926 |
| Forced Medium Reliability x Free Low Reliability | 64.24 | 37.4 | 33 | 1.716 | 0.5314 |
| Forced Medium Reliability x Forced High Reliability | **196.23** | **20.2** | **33** | **9.734** | **<.0001** |
| Forced Medium Reliability x Free High Reliability | **156.26** | **36.6** | **33** | **4.265** | **0.0020** |
| Free Medium Reliability x Forced Low Reliability | -61.51 | 29.5 | 33 | -2.086 | 0.3191 |
| Free Medium Reliability x Free Low Reliability | 12.63 | 20.0 | 33 | 0.633 | 0.9877 |
| Free Medium Reliability x Forced High Reliability | 144.62 | 32.1 | 33 | 4.507 | 0.0010 |
| Free Medium Reliability x Free High Reliability | **104.64** | **17.9** | **33** | **5.839** | **<.0001** |
| Forced Low Reliability x Free Low Reliability | 74.13 | 34.0 | 33 | 2.180 | 0.2737 |
| Forced Low Reliability x Forced High Reliability | **206.12** | **23.9** | **33** | **8.632** | **<.0001** |
| Forced Low Reliability x Free High Reliability | **166.15** | **35.8** | **33** | **4.642** | **0.0007** |
| Free Low Reliability x Forced High Reliability | **131.99** | **36.3** | **33** | **3.637** | **0.0110** |
| Free Low Reliability x Free High Reliability | **92.01** | **24.3** | **33** | **3.793** | **0.0073** |
| Forced High Reliability x Free High Reliability | -39.98 | 30.0 | 33 | -1.331 | 0.7660 |

**Table of Bonferroni Analyses – Explicability x Difficulty x Reliability**

| Condition tested | Estimate | Standard Error | DF | t-ratio | P-value |
| --- | --- | --- | --- | --- | --- |
| UnGuided Easy Medium Reliability x Guided Easy Medium Reliability | -58.22 | 38.7 | 33 | -1.503 | 0.9292 |
| UnGuided Easy Medium Reliability x UnGuided Hard Medium Reliability | **-181.36** | **43.7** | **33** | **-4.148** | **0.0101** |
| UnGuided Easy Medium Reliability x Guided Hard Medium Reliability | **-412.05** | **71.2** | **33** | **-5.791** | **0.0001** |
| UnGuided Easy Medium Reliability x UnGuided Easy Low Reliability | 50.72 | 36.9 | 33 | 1.376 | 0.9603 |
| UnGuided Easy Medium Reliability x Guided Easy Low Reliability | **-159.92** | **42.4** | **33** | **-3.776** | **0.0262** |
| UnGuided Easy Medium Reliability x UnGuided Hard Low Reliability | -155.96 | 50.3 | 33 | -3.098 | 0.1256 |
| UnGuided Easy Medium Reliability x Guided Hard Low Reliability | **-381.00** | **53.1** | **33** | **-7.176** | **<.0001** |
| UnGuided Easy Medium Reliability x UnGuided Easy High Reliability | **148.86** | **33.1** | **33** | **4.499** | **0.0039** |
| UnGuided Easy Medium Reliability x Guided Easy High Reliability | 116.47 | 44.3 | 33 | 2.631 | 0.3038 |
| UnGuided Easy Medium Reliability x UnGuided Hard High Reliability | -122.94 | 51.4 | 33 | -2.392 | 0.4382 |
| UnGuided Easy Medium Reliability x Guided Hard High Reliability | -192.28 | 56.6 | 33 | -3.397 | 0.0652 |
| Guided Easy Medium Reliability x UnGuided Hard Medium Reliability | -123.15 | 41.3 | 33 | -2.985 | 0.1584 |
| Guided Easy Medium Reliability x Guided Hard Medium Reliability | **-353.83** | **51.7** | **33** | **-6.848** | **<.0001** |
| Guided Easy Medium Reliability x UnGuided Easy Low Reliability | **108.94** | **25.3** | **33** | **4.298** | **0.0067** |
| Guided Easy Medium Reliability x Guided Easy Low Reliability | **-101.70** | **28.2** | **33** | **-3.601** | **0.0404** |
| Guided Easy Medium Reliability x UnGuided Hard Low Reliability | -97.74 | 43.9 | 33 | -2.227 | 0.5430 |
| Guided Easy Medium Reliability x Guided Hard Low Reliability | **-322.78** | **38.8** | **33** | **-8.309** | **<.0001** |
| Guided Easy Medium Reliability x UnGuided Easy High Reliability | **207.08** | **33.3** | **33** | **6.223** | **<.0001** |
| Guided Easy Medium Reliability x Guided Easy High Reliability | **174.69** | **30.6** | **33** | **5.716** | **0.0001** |
| Guided Easy Medium Reliability x UnGuided Hard High Reliability | -64.72 | 45.0 | 33 | -1.438 | 0.9468 |
| Guided Easy Medium Reliability x Guided Hard High Reliability | -134.06 | 39.7 | 33 | -3.379 | 0.0680 |
| UnGuided Hard Medium Reliability x Guided Hard Medium Reliability | **-230.69** | **52.1** | **33** | **-4.427** | **0.0048** |
| UnGuided Hard Medium Reliability x UnGuided Easy Low Reliability | **232.09** | **39.8** | **33** | **5.828** | **0.0001** |
| UnGuided Hard Medium Reliability x Guided Easy Low Reliability | 21.44 | 49.6 | 33 | 0.432 | 1.0000 |
| UnGuided Hard Medium Reliability x UnGuided Hard Low Reliability | 25.41 | 32.5 | 33 | 0.781 | 0.9996 |
| UnGuided Hard Medium Reliability x Guided Hard Low Reliability | **-199.63** | **38.8** | **33** | **-5.144** | **0.0006** |
| UnGuided Hard Medium Reliability x UnGuided Easy High Reliability | **330.23** | **45.9** | **33** | **7.193** | **<.0001** |
| UnGuided Hard Medium Reliability x Guided Easy High Reliability | **297.84** | **46.7** | **33** | **6.383** | **<.0001** |
| UnGuided Hard Medium Reliability x UnGuided Hard High Reliability | 58.43 | 31.7 | 33 | 1.841 | 0.7847 |
| UnGuided Hard Medium Reliability x Guided Hard High Reliability | -10.91 | 42.4 | 33 | -0.258 | 1.0000 |
| Guided Hard Medium Reliability x UnGuided Easy Low Reliability | **462.77** | **60.8** | **33** | **7.606** | **<.0001** |
| Guided Hard Medium Reliability x Guided Easy Low Reliability | **252.13** | **61.3** | **33** | **4.116** | **0.0110** |
| Guided Hard Medium Reliability x UnGuided Hard Low Reliability | **256.09** | **42.5** | **33** | **6.030** | **0.0001** |
| Guided Hard Medium Reliability x Guided Hard Low Reliability | 31.05 | 46.6 | 33 | 0.666 | 0.9999 |
| Guided Hard Medium Reliability x UnGuided Easy High Reliability | **560.91** | **70.1** | **33** | **8.007** | **<.0001** |
| Guided Hard Medium Reliability x Guided Easy High Reliability | **528.52** | **60.2** | **33** | **8.772** | **<.0001** |
| Guided Hard Medium Reliability x UnGuided Hard High Reliability | **289.11** | **40.5** | **33** | **7.144** | **<.0001** |
| Guided Hard Medium Reliability x Guided Hard High Reliability | **219.77** | **41.3** | **33** | **5.327** | **0.0004** |
| UnGuided Easy Low Reliability x Guided Easy Low Reliability | **-210.64** | **37.1** | **33** | **-5.675** | **0.0001** |
| UnGuided Easy Low Reliability x UnGuided Hard Low Reliability | **-206.68** | **48.4** | **33** | **-4.272** | **0.0072** |
| UnGuided Easy Low Reliability x Guided Hard Low Reliability | **-431.72** | **47.0** | **33** | **-9.179** | **<.0001** |
| UnGuided Easy Low Reliability x UnGuided Easy High Reliability | 98.14 | 30.6 | 33 | 3.205 | 0.1002 |
| UnGuided Easy Low Reliability x Guided Easy High Reliability | 65.75 | 26.7 | 33 | 2.460 | 0.3974 |
| UnGuided Easy Low Reliability x UnGuided Hard High Reliability | **-173.66** | **49.3** | **33** | **-3.519** | **0.0490** |
| UnGuided Easy Low Reliability x Guided Hard High Reliability | **-243.00** | **42.4** | **33** | **-5.732** | **0.0001** |
| Guided Easy Low Reliability x UnGuided Hard Low Reliability | 3.96 | 58.9 | 33 | 0.067 | 1.0000 |
| Guided Easy Low Reliability x Guided Hard Low Reliability | **-221.08** | **36.6** | **33** | **-6.045** | **<.0001** |
| Guided Easy Low Reliability x UnGuided Easy High Reliability | **308.78** | **38.3** | **33** | **8.071** | **<.0001** |
| Guided Easy Low Reliability x Guided Easy High Reliability | **276.39** | **44.9** | **33** | **6.154** | **<.0001** |
| Guided Easy Low Reliability x UnGuided Hard High Reliability | 36.98 | 53.4 | 33 | 0.693 | 0.9999 |
| Guided Easy Low Reliability x Guided Hard High Reliability | -32.36 | 53.4 | 33 | -0.606 | 1.0000 |
| UnGuided Hard Low Reliability x Guided Hard Low Reliability | **-225.04** | **46.5** | **33** | **-4.840** | **0.0015** |
| UnGuided Hard Low Reliability x UnGuided Easy High Reliability | **304.82** | **56.0** | **33** | **5.448** | **0.0003** |
| UnGuided Hard Low Reliability x Guided Easy High Reliability | **272.43** | **53.8** | **33** | **5.067** | **0.0008** |
| UnGuided Hard Low Reliability x UnGuided Hard High Reliability | 33.02 | 28.8 | 33 | 1.146 | 0.9897 |
| UnGuided Hard Low Reliability x Guided Hard High Reliability | **-36.32** | **38.9** | **33** | **-0.934** | **0.9981** |
| Guided Hard Low Reliability x UnGuided Easy High Reliability | **529.86** | **52.5** | **33** | **10.087** | **<.0001** |
| Guided Hard Low Reliability x Guided Easy High Reliability | **497.47** | **56.0** | **33** | **8.890** | **<.0001** |
| Guided Hard Low Reliability x UnGuided Hard High Reliability | **258.06** | **39.7** | **33** | **6.501** | **<.0001** |
| Guided Hard Low Reliability x Guided Hard High Reliability | **188.72** | **47.5** | **33** | **3.969** | **0.0161** |
| UnGuided Easy High Reliability x Guided Easy High Reliability | -32.39 | 39.4 | 33 | -0.822 | 0.9994 |
| UnGuided Easy High Reliability x UnGuided Hard High Reliability | **-271.80** | **54.8** | **33** | **-4.961** | **0.0011** |
| UnGuided Easy High Reliability x Guided Hard High Reliability | **-341.14** | **52.4** | **33** | **-6.514** | **<.0001** |
| Guided Easy High Reliability x UnGuided Hard High Reliability | **-239.41** | **52.0** | **33** | **-4.605** | **0.0029** |
| Guided Easy High Reliability x Guided Hard High Reliability | **-308.75** | **40.9** | **33** | **-7.542** | **<.0001** |
| UnGuided Hard High Reliability x Guided Hard High Reliability | -69.34 | 42.3 | 33 | -1.638 | 0.8823 |

**Table of Bonferroni Analyses – Level of Automation x Difficulty x Reliability x Explicability**

| Condition tested | Estimate | Standard Error | DF | t-ratio | P-value |
| --- | --- | --- | --- | --- | --- |
| Forced Easy Medium Reliability UnGuided x Free Easy Medium Reliability UnGuided | **201.72** | **43.8** | **33** | **4.607** | **0.0096** |
| Forced Easy Medium Reliability UnGuided x Forced Hard Medium Reliability UnGuided | -128.44 | 52.8 | 33 | -2.433 | 0.7120 |
| Forced Easy Medium Reliability UnGuided x Free Hard Medium Reliability UnGuided | -32.56 | 48.9 | 33 | -0.666 | 1.0000 |
| Forced Easy Medium Reliability UnGuided x Forced Easy Low Reliability UnGuided | 54.53 | 40.0 | 33 | 1.363 | 0.9989 |
| Forced Easy Medium Reliability UnGuided x Free Easy Low Reliability UnGuided | **248.63** | **46.8** | **33** | **5.314** | **0.0014** |
| Forced Easy Medium Reliability UnGuided x Forced Hard Low Reliability UnGuided | -63.96 | 50.0 | 33 | -1.280 | 0.9996 |
| Forced Easy Medium Reliability UnGuided x Free Hard Low Reliability UnGuided | -46.24 | 67.9 | 33 | -0.681 | 1.0000 |
| Forced Easy Medium Reliability UnGuided x Forced Easy High Reliability UnGuided | **261.63** | **43.4** | **33** | **6.025** | **0.0002** |
| Forced Easy Medium Reliability UnGuided x Free Easy High Reliability UnGuided | **237.82** | **51.5** | **33** | **4.617** | **0.0094** |
| Forced Easy Medium Reliability UnGuided x Forced Hard High Reliability UnGuided | -60.55 | 59.9 | 33 | -1.011 | 1.0000 |
| Forced Easy Medium Reliability UnGuided x Free Hard High Reliability UnGuided | 16.40 | 58.1 | 33 | 0.282 | 1.0000 |
| Forced Easy Medium Reliability UnGuided x Forced Easy Medium Reliability Guided | -3.61 | 40.9 | 33 | -0.088 | 1.0000 |
| Forced Easy Medium Reliability UnGuided x Free Easy Medium Reliability Guided | 88.90 | 54.2 | 33 | 1.642 | 0.9890 |
| Forced Easy Medium Reliability UnGuided x Forced Hard Medium Reliability Guided | -219.36 | 66.8 | 33 | -3.286 | 0.2115 |
| Forced Easy Medium Reliability UnGuided x Free Hard Medium Reliability Guided | **-403.01** | **85.8** | **33** | **-4.695** | **0.0076** |
| Forced Easy Medium Reliability UnGuided x Forced Easy Low Reliability Guided | -102.56 | 45.9 | 33 | -2.236 | 0.8254 |
| Forced Easy Medium Reliability UnGuided x Free Easy Low Reliability Guided | -15.56 | 59.6 | 33 | -0.261 | 1.0000 |
| Forced Easy Medium Reliability UnGuided x Forced Hard Low Reliability Guided | **-278.99** | **50.3** | **33** | **-5.548** | **0.0007** |
| Forced Easy Medium Reliability UnGuided x Free Hard Low Reliability Guided | -281.28 | 74.5 | 33 | -3.774 | 0.0761 |
| Forced Easy Medium Reliability UnGuided x Forced Easy High Reliability Guided | **228.16** | **46.9** | **33** | **4.865** | **0.0048** |
| Forced Easy Medium Reliability UnGuided x Free Easy High Reliability Guided | 206.52 | 53.6 | 33 | 3.852 | 0.0635 |
| Forced Easy Medium Reliability UnGuided x Forced Hard High Reliability Guided | 4.29 | 50.6 | 33 | 0.085 | 1.0000 |
| Forced Easy Medium Reliability UnGuided x Free Hard High Reliability Guided | -187.12 | 68.5 | 33 | -2.733 | 0.5141 |
| Free Easy Medium Reliability UnGuided x Forced Hard Medium Reliability UnGuided | **-330.16** | **71.2** | **33** | **-4.638** | **0.0089** |
| Free Easy Medium Reliability UnGuided x Free Hard Medium Reliability UnGuided | **-234.28** | **47.8** | **33** | **-4.897** | **0.0044** |
| Free Easy Medium Reliability UnGuided x Forced Easy Low Reliability UnGuided | -147.19 | 50.6 | 33 | -2.910 | 0.4013 |
| Free Easy Medium Reliability UnGuided x Free Easy Low Reliability UnGuided | 46.91 | 46.0 | 33 | 1.019 | 1.0000 |
| Free Easy Medium Reliability UnGuided x Forced Hard Low Reliability UnGuided | -265.68 | 68.4 | 33 | -3.886 | 0.0588 |
| Free Easy Medium Reliability UnGuided x Free Hard Low Reliability UnGuided | -247.96 | 64.1 | 33 | -3.868 | 0.0613 |
| Free Easy Medium Reliability UnGuided x Forced Easy High Reliability UnGuided | 59.91 | 51.4 | 33 | 1.165 | 0.9999 |
| Free Easy Medium Reliability UnGuided x Free Easy High Reliability UnGuided | 36.10 | 38.5 | 33 | 0.939 | 1.0000 |
| Free Easy Medium Reliability UnGuided x Forced Hard High Reliability UnGuided | -262.27 | 71.6 | 33 | -3.666 | 0.0969 |
| Free Easy Medium Reliability UnGuided x Free Hard High Reliability UnGuided | -185.32 | 65.1 | 33 | -2.846 | 0.4409 |
| Free Easy Medium Reliability UnGuided x Forced Easy Medium Reliability Guided | **-205.33** | **48.9** | **33** | **-4.203** | **0.0272** |
| Free Easy Medium Reliability UnGuided x Free Easy Medium Reliability Guided | -112.82 | 50.5 | 33 | -2.236 | 0.8256 |
| Free Easy Medium Reliability UnGuided x Forced Hard Medium Reliability Guided | **-421.08** | **73.8** | **33** | **-5.704** | **0.0005** |
| Free Easy Medium Reliability UnGuided x Free Hard Medium Reliability Guided | **-604.73** | **92.5** | **33** | **-6.537** | **<.0001** |
| Free Easy Medium Reliability UnGuided x Forced Easy Low Reliability Guided | **-304.28** | **46.5** | **33** | **-6.545** | **<.0001** |
| Free Easy Medium Reliability UnGuided x Free Easy Low Reliability Guided | -217.28 | 63.7 | 33 | -3.410 | 0.1661 |
| Free Easy Medium Reliability UnGuided x Forced Hard Low Reliability Guided | **-480.72** | **58.2** | **33** | **-8.259** | **<.0001** |
| Free Easy Medium Reliability UnGuided x Free Hard Low Reliability Guided | **-483.00** | **78.5** | **33** | **-6.154** | **0.0001** |
| Free Easy Medium Reliability UnGuided x Forced Easy High Reliability Guided | 26.43 | 50.1 | 33 | 0.528 | 1.0000 |
| Free Easy Medium Reliability UnGuided x Free Easy High Reliability Guided | 4.79 | 56.0 | 33 | 0.086 | 1.0000 |
| Free Easy Medium Reliability UnGuided x Forced Hard High Reliability Guided | -197.43 | 68.9 | 33 | -2.864 | 0.4298 |
| Free Easy Medium Reliability UnGuided x Free Hard High Reliability Guided | **-388.84** | **73.0** | **33** | **-5.328** | **0.0013** |
| Forced Hard Medium Reliability UnGuided x Free Hard Medium Reliability UnGuided | 95.88 | 54.5 | 33 | 1.758 | 0.9769 |
| Forced Hard Medium Reliability UnGuided x Forced Easy Low Reliability UnGuided | 182.98 | 49.6 | 33 | 3.689 | 0.0920 |
| Forced Hard Medium Reliability UnGuided x Free Easy Low Reliability UnGuided | **377.07** | **65.4** | **33** | **5.762** | **0.0004** |
| Forced Hard Medium Reliability UnGuided x Forced Hard Low Reliability UnGuided | 64.49 | 39.9 | 33 | 1.616 | 0.9908 |
| Forced Hard Medium Reliability UnGuided x Free Hard Low Reliability UnGuided | 82.20 | 62.6 | 33 | 1.314 | 0.9994 |
| Forced Hard Medium Reliability UnGuided x Forced Easy High Reliability UnGuided | **390.07** | **53.2** | **33** | **7.330** | **<.0001** |
| Forced Hard Medium Reliability UnGuided x Free Easy High Reliability UnGuided | **366.26** | **78.8** | **33** | **4.649** | **0.0086** |
| Forced Hard Medium Reliability UnGuided x Forced Hard High Reliability UnGuided | 67.89 | 44.7 | 33 | 1.518 | 0.9956 |
| Forced Hard Medium Reliability UnGuided x Free Hard High Reliability UnGuided | 144.84 | 57.4 | 33 | 2.522 | 0.6544 |
| Forced Hard Medium Reliability UnGuided x Forced Easy Medium Reliability Guided | 124.83 | 51.8 | 33 | 2.412 | 0.7250 |
| Forced Hard Medium Reliability UnGuided x Free Easy Medium Reliability Guided | 217.34 | 62.9 | 33 | 3.456 | 0.1514 |
| Forced Hard Medium Reliability UnGuided x Forced Hard Medium Reliability Guided | -90.92 | 45.3 | 33 | -2.005 | 0.9222 |
| Forced Hard Medium Reliability UnGuided x Free Hard Medium Reliability Guided | -274.57 | 77.7 | 33 | -3.533 | 0.1290 |
| Forced Hard Medium Reliability UnGuided x Forced Easy Low Reliability Guided | 25.88 | 61.2 | 33 | 0.423 | 1.0000 |
| Forced Hard Medium Reliability UnGuided x Free Easy Low Reliability Guided | 112.89 | 71.8 | 33 | 1.573 | 0.9933 |
| Forced Hard Medium Reliability UnGuided x Forced Hard Low Reliability Guided | -150.55 | 48.6 | 33 | -3.096 | 0.2979 |
| Forced Hard Medium Reliability UnGuided x Free Hard Low Reliability Guided | -152.84 | 73.3 | 33 | -2.085 | 0.8937 |
| Forced Hard Medium Reliability UnGuided x Forced Easy High Reliability Guided | **356.60** | **61.6** | **33** | **5.785** | **0.0004** |
| Forced Hard Medium Reliability UnGuided x Free Easy High Reliability Guided | **334.96** | **60.2** | **33** | **5.568** | **0.0007** |
| Forced Hard Medium Reliability UnGuided x Forced Hard High Reliability Guided | 132.73 | 44.2 | 33 | 3.003 | 0.3472 |
| Forced Hard Medium Reliability UnGuided x Free Hard High Reliability Guided | -58.68 | 66.3 | 33 | -0.885 | 1.0000 |
| Free Hard Medium Reliability UnGuided x Forced Easy Low Reliability UnGuided | 87.10 | 45.0 | 33 | 1.936 | 0.9423 |
| Free Hard Medium Reliability UnGuided x Free Easy Low Reliability UnGuided | **281.20** | **40.8** | **33** | **6.893** | **<.0001** |
| Free Hard Medium Reliability UnGuided x Forced Hard Low Reliability UnGuided | -31.39 | 60.0 | 33 | -0.523 | 1.0000 |
| Free Hard Medium Reliability UnGuided x Free Hard Low Reliability UnGuided | -13.68 | 44.1 | 33 | -0.310 | 1.0000 |
| Free Hard Medium Reliability UnGuided x Forced Easy High Reliability UnGuided | **294.19** | **45.2** | **33** | **6.508** | **<.0001** |
| Free Hard Medium Reliability UnGuided x Free Easy High Reliability UnGuided | **270.38** | **51.8** | **33** | **5.218** | **0.0018** |
| Free Hard Medium Reliability UnGuided x Forced Hard High Reliability UnGuided | -27.99 | 62.1 | 33 | -0.450 | 1.0000 |
| Free Hard Medium Reliability UnGuided x Free Hard High Reliability UnGuided | 48.97 | 41.0 | 33 | 1.194 | 0.9998 |
| Free Hard Medium Reliability UnGuided x Forced Easy Medium Reliability Guided | 28.95 | 47.3 | 33 | 0.613 | 1.0000 |
| Free Hard Medium Reliability UnGuided x Free Easy Medium Reliability Guided | 121.46 | 50.4 | 33 | 2.411 | 0.7253 |
| Free Hard Medium Reliability UnGuided x Forced Hard Medium Reliability Guided | -186.80 | 60.1 | 33 | -3.106 | 0.2925 |
| Free Hard Medium Reliability UnGuided x Free Hard Medium Reliability Guided | **-370.45** | **76.0** | **33** | **-4.871** | **0.0047** |
| Free Hard Medium Reliability UnGuided x Forced Easy Low Reliability Guided | -70.00 | 50.8 | 33 | -1.379 | 0.9988 |
| Free Hard Medium Reliability UnGuided x Free Easy Low Reliability Guided | 17.01 | 64.2 | 33 | 0.265 | 1.0000 |
| Free Hard Medium Reliability UnGuided x Forced Hard Low Reliability Guided | **-246.43** | **52.9** | **33** | **-4.662** | **0.0083** |
| Free Hard Medium Reliability UnGuided x Free Hard Low Reliability Guided | **-248.72** | **53.9** | **33** | **-4.613** | **0.0095** |
| Free Hard Medium Reliability UnGuided x Forced Easy High Reliability Guided | **260.72** | **50.8** | **33** | **5.130** | **0.0023** |
| Free Hard Medium Reliability UnGuided x Free Easy High Reliability Guided | **239.08** | **51.3** | **33** | **4.657** | **0.0084** |
| Free Hard Medium Reliability UnGuided x Forced Hard High Reliability Guided | 36.85 | 57.9 | 33 | 0.636 | 1.0000 |
| Free Hard Medium Reliability UnGuided x Free Hard High Reliability Guided | -154.56 | 55.2 | 33 | -2.799 | 0.4710 |
| Forced Easy Low Reliability UnGuided x Free Easy Low Reliability UnGuided | **194.10** | **33.4** | **33** | **5.811** | **0.0003** |
| Forced Easy Low Reliability UnGuided x Forced Hard Low Reliability UnGuided | -118.49 | 50.1 | 33 | -2.365 | 0.7534 |
| Forced Easy Low Reliability UnGuided x Free Hard Low Reliability UnGuided | -100.77 | 61.0 | 33 | -1.651 | 0.9883 |
| Forced Easy Low Reliability UnGuided x Forced Easy High Reliability UnGuided | **207.09** | **35.9** | **33** | **5.766** | **0.0004** |
| Forced Easy Low Reliability UnGuided x Free Easy High Reliability UnGuided | 183.28 | 53.2 | 33 | 3.442 | 0.1556 |
| Forced Easy Low Reliability UnGuided x Forced Hard High Reliability UnGuided | -115.09 | 62.0 | 33 | -1.858 | 0.9604 |
| Forced Easy Low Reliability UnGuided x Free Hard High Reliability UnGuided | -38.13 | 54.3 | 33 | -0.702 | 1.0000 |
| Forced Easy Low Reliability UnGuided x Forced Easy Medium Reliability Guided | -58.15 | 26.6 | 33 | -2.186 | 0.8502 |
| Forced Easy Low Reliability UnGuided x Free Easy Medium Reliability Guided | 34.37 | 42.6 | 33 | 0.807 | 1.0000 |
| Forced Easy Low Reliability UnGuided x Forced Hard Medium Reliability Guided | **-273.90** | **60.1** | **33** | **-4.560** | **0.0109** |
| Forced Easy Low Reliability UnGuided x Free Hard Medium Reliability Guided | **-457.55** | **75.0** | **33** | **-6.100** | **0.0002** |
| Forced Easy Low Reliability UnGuided x Forced Easy Low Reliability Guided | -157.10 | 42.5 | 33 | -3.695 | 0.0909 |
| Forced Easy Low Reliability UnGuided x Free Easy Low Reliability Guided | -70.09 | 51.5 | 33 | -1.360 | 0.9990 |
| Forced Easy Low Reliability UnGuided x Forced Hard Low Reliability Guided | **-333.53** | **53.5** | **33** | **-6.233** | **0.0001** |
| Forced Easy Low Reliability UnGuided x Free Hard Low Reliability Guided | **-335.81** | **61.4** | **33** | **-5.467** | **0.0009** |
| Forced Easy Low Reliability UnGuided x Forced Easy High Reliability Guided | **173.62** | **29.8** | **33** | **5.825** | **0.0003** |
| Forced Easy Low Reliability UnGuided x Free Easy High Reliability Guided | 151.98 | 44.4 | 33 | 3.424 | 0.1614 |
| Forced Easy Low Reliability UnGuided x Forced Hard High Reliability Guided | -50.25 | 46.0 | 33 | -1.094 | 1.0000 |
| Forced Easy Low Reliability UnGuided x Free Hard High Reliability Guided | **-241.66** | **52.8** | **33** | **-4.580** | **0.0103** |
| Free Easy Low Reliability UnGuided x Forced Hard Low Reliability UnGuided | **-312.59** | **65.0** | **33** | **-4.810** | **0.0056** |
| Free Easy Low Reliability UnGuided x Free Hard Low Reliability UnGuided | **-294.87** | **62.1** | **33** | **-4.746** | **0.0066** |
| Free Easy Low Reliability UnGuided x Forced Easy High Reliability UnGuided | 13.00 | 39.5 | 33 | 0.329 | 1.0000 |
| Free Easy Low Reliability UnGuided x Free Easy High Reliability UnGuided | -10.81 | 39.3 | 33 | -0.275 | 1.0000 |
| Free Easy Low Reliability UnGuided x Forced Hard High Reliability UnGuided | **-309.18** | **71.7** | **33** | **-4.313** | **0.0207** |
| Free Easy Low Reliability UnGuided x Free Hard High Reliability UnGuided | **-232.23** | **52.6** | **33** | **-4.414** | **0.0159** |
| Free Easy Low Reliability UnGuided x Forced Easy Medium Reliability Guided | **-252.24** | **34.5** | **33** | **-7.306** | **<.0001** |
| Free Easy Low Reliability UnGuided x Free Easy Medium Reliability Guided | **-159.73** | **40.0** | **33** | **-3.997** | **0.0452** |
| Free Easy Low Reliability UnGuided x Forced Hard Medium Reliability Guided | **-467.99** | **70.4** | **33** | **-6.648** | **<.0001** |
| Free Easy Low Reliability UnGuided x Free Hard Medium Reliability Guided | **-651.65** | **73.4** | **33** | **-8.873** | **<.0001** |
| Free Easy Low Reliability UnGuided x Forced Easy Low Reliability Guided | **-351.19** | **44.2** | **33** | **-7.942** | **<.0001** |
| Free Easy Low Reliability UnGuided x Free Easy Low Reliability Guided | **-264.19** | **54.7** | **33** | **-4.830** | **0.0053** |
| Free Easy Low Reliability UnGuided x Forced Hard Low Reliability Guided | **-527.63** | **64.3** | **33** | **-8.209** | **<.0001** |
| Free Easy Low Reliability UnGuided x Free Hard Low Reliability Guided | **-529.91** | **60.3** | **33** | **-8.787** | **<.0001** |
| Free Easy Low Reliability UnGuided x Forced Easy High Reliability Guided | -20.48 | 28.2 | 33 | -0.725 | 1.0000 |
| Free Easy Low Reliability UnGuided x Free Easy High Reliability Guided | -42.12 | 35.7 | 33 | -1.180 | 0.9999 |
| Free Easy Low Reliability UnGuided x Forced Hard High Reliability Guided | **-244.35** | **57.3** | **33** | **-4.261** | **0.0236** |
| Free Easy Low Reliability UnGuided x Free Hard High Reliability Guided | **-435.75** | **52.2** | **33** | **-8.354** | **<.0001** |
| Forced Hard Low Reliability UnGuided x Free Hard Low Reliability UnGuided | 17.72 | 62.0 | 33 | 0.286 | 1.0000 |
| Forced Hard Low Reliability UnGuided x Forced Easy High Reliability UnGuided | **325.59** | **63.6** | **33** | **5.116** | **0.0024** |
| Forced Hard Low Reliability UnGuided x Free Easy High Reliability UnGuided | **301.77** | **74.1** | **33** | **4.074** | **0.0375** |
| Forced Hard Low Reliability UnGuided x Forced Hard High Reliability UnGuided | 3.40 | 37.3 | 33 | 0.091 | 1.0000 |
| Forced Hard Low Reliability UnGuided x Free Hard High Reliability UnGuided | 80.36 | 59.1 | 33 | 1.359 | 0.9990 |
| Forced Hard Low Reliability UnGuided x Forced Easy Medium Reliability Guided | 60.34 | 50.1 | 33 | 1.206 | 0.9998 |
| Forced Hard Low Reliability UnGuided x Free Easy Medium Reliability Guided | 152.86 | 60.3 | 33 | 2.535 | 0.6460 |
| Forced Hard Low Reliability UnGuided x Forced Hard Medium Reliability Guided | -155.41 | 41.3 | 33 | -3.761 | 0.0784 |
| Forced Hard Low Reliability UnGuided x Free Hard Medium Reliability Guided | **-339.06** | **70.3** | **33** | **-4.820** | **0.0054** |
| Forced Hard Low Reliability UnGuided x Forced Easy Low Reliability Guided | -38.61 | 57.3 | 33 | -0.674 | 1.0000 |
| Forced Hard Low Reliability UnGuided x Free Easy Low Reliability Guided | 48.40 | 77.5 | 33 | 0.624 | 1.0000 |
| Forced Hard Low Reliability UnGuided x Forced Hard Low Reliability Guided | -215.04 | 58.1 | 33 | -3.703 | 0.0892 |
| Forced Hard Low Reliability UnGuided x Free Hard Low Reliability Guided | -217.32 | 72.7 | 33 | -2.989 | 0.3553 |
| Forced Hard Low Reliability UnGuided x Forced Easy High Reliability Guided | **292.11** | **58.4** | **33** | **5.000** | **0.0033** |
| Forced Hard Low Reliability UnGuided x Free Easy High Reliability Guided | **270.47** | **60.4** | **33** | **4.477** | **0.0136** |
| Forced Hard Low Reliability UnGuided x Forced Hard High Reliability Guided | 68.24 | 39.7 | 33 | 1.721 | 0.9816 |
| Forced Hard Low Reliability UnGuided x Free Hard High Reliability Guided | -123.16 | 60.0 | 33 | -2.051 | 0.9064 |
| Free Hard Low Reliability UnGuided x Forced Easy High Reliability UnGuided | **307.87** | **65.7** | **33** | **4.683** | **0.0079** |
| Free Hard Low Reliability UnGuided x Free Easy High Reliability UnGuided | **284.06** | **69.8** | **33** | **4.071** | **0.0378** |
| Free Hard Low Reliability UnGuided x Forced Hard High Reliability UnGuided | -14.31 | 66.0 | 33 | -0.217 | 1.0000 |
| Free Hard Low Reliability UnGuided x Free Hard High Reliability UnGuided | 62.64 | 41.9 | 33 | 1.496 | 0.9964 |
| Free Hard Low Reliability UnGuided x Forced Easy Medium Reliability Guided | 42.63 | 61.8 | 33 | 0.690 | 1.0000 |
| Free Hard Low Reliability UnGuided x Free Easy Medium Reliability Guided | 135.14 | 56.6 | 33 | 2.386 | 0.7409 |
| Free Hard Low Reliability UnGuided x Forced Hard Medium Reliability Guided | -173.12 | 55.1 | 33 | -3.141 | 0.2755 |
| Free Hard Low Reliability UnGuided x Free Hard Medium Reliability Guided | **-356.78** | **70.9** | **33** | **-5.034** | **0.0030** |
| Free Hard Low Reliability UnGuided x Forced Easy Low Reliability Guided | -56.32 | 65.9 | 33 | -0.855 | 1.0000 |
| Free Hard Low Reliability UnGuided x Free Easy Low Reliability Guided | 30.68 | 82.8 | 33 | 0.371 | 1.0000 |
| Free Hard Low Reliability UnGuided x Forced Hard Low Reliability Guided | -232.76 | 66.6 | 33 | -3.494 | 0.1400 |
| Free Hard Low Reliability UnGuided x Free Hard Low Reliability Guided | -235.04 | 61.9 | 33 | -3.798 | 0.0721 |
| Free Hard Low Reliability UnGuided x Forced Easy High Reliability Guided | **274.39** | **68.2** | **33** | **4.025** | **0.0422** |
| Free Hard Low Reliability UnGuided x Free Easy High Reliability Guided | 252.75 | 68.2 | 33 | 3.707 | 0.0885 |
| Free Hard Low Reliability UnGuided x Forced Hard High Reliability Guided | 50.53 | 63.4 | 33 | 0.797 | 1.0000 |
| Free Hard Low Reliability UnGuided x Free Hard High Reliability Guided | -140.88 | 57.4 | 33 | -2.454 | 0.6983 |
| Forced Easy High Reliability UnGuided x Free Easy High Reliability UnGuided | -23.81 | 48.6 | 33 | -0.490 | 1.0000 |
| Forced Easy High Reliability UnGuided x Forced Hard High Reliability UnGuided | -322.18 | 67.9 | 33 | -4.743 | 0.0067 |
| Forced Easy High Reliability UnGuided x Free Hard High Reliability UnGuided | **-245.23** | **57.3** | **33** | **-4.278** | **0.0226** |
| Forced Easy High Reliability UnGuided x Forced Easy Medium Reliability Guided | **-265.24** | **43.8** | **33** | **-6.052** | **0.0002** |
| Forced Easy High Reliability UnGuided x Free Easy Medium Reliability Guided | -172.73 | 44.8 | 33 | -3.860 | 0.0625 |
| Forced Easy High Reliability UnGuided x Forced Hard Medium Reliability Guided | **-480.99** | **68.4** | **33** | **-7.028** | **<.0001** |
| Forced Easy High Reliability UnGuided x Free Hard Medium Reliability Guided | **-664.64** | **87.0** | **33** | **-7.639** | **<.0001** |
| Forced Easy High Reliability UnGuided x Forced Easy Low Reliability Guided | **-364.19** | **50.5** | **33** | **-7.212** | **<.0001** |
| Forced Easy High Reliability UnGuided x Free Easy Low Reliability Guided | **-277.18** | **50.6** | **33** | **-5.474** | **0.0009** |
| Forced Easy High Reliability UnGuided x Forced Hard Low Reliability Guided | **-540.62** | **55.5** | **33** | **-9.739** | **<.0001** |
| Forced Easy High Reliability UnGuided x Free Hard Low Reliability Guided | **-542.91** | **67.0** | **33** | **-8.106** | **<.0001** |
| Forced Easy High Reliability UnGuided x Forced Easy High Reliability Guided | -33.47 | 46.7 | 33 | -0.718 | 1.0000 |
| Forced Easy High Reliability UnGuided x Free Easy High Reliability Guided | -55.11 | 50.4 | 33 | -1.094 | 1.0000 |
| Forced Easy High Reliability UnGuided x Forced Hard High Reliability Guided | **-257.34** | **54.7** | **33** | **-4.708** | **0.0074** |
| Forced Easy High Reliability UnGuided x Free Hard High Reliability Guided | **-448.75** | **63.5** | **33** | **-7.070** | **<.0001** |
| Free Easy High Reliability UnGuided x Forced Hard High Reliability UnGuided | **-298.37** | **76.9** | **33** | **-3.879** | **0.0597** |
| Free Easy High Reliability UnGuided x Free Hard High Reliability UnGuided | -221.42 | 66.3 | 33 | -3.341 | 0.1903 |
| Free Easy High Reliability UnGuided x Forced Easy Medium Reliability Guided | **-241.43** | **51.5** | **33** | **-4.684** | **0.0078** |
| Free Easy High Reliability UnGuided x Free Easy Medium Reliability Guided | -148.92 | 42.9 | 33 | -3.474 | 0.1458 |
| Free Easy High Reliability UnGuided x Forced Hard Medium Reliability Guided | **-457.18** | **79.5** | **33** | **-5.751** | **0.0004** |
| Free Easy High Reliability UnGuided x Free Hard Medium Reliability Guided | **-640.83** | **84.1** | **33** | **-7.616** | **<.0001** |
| Free Easy High Reliability UnGuided x Forced Easy Low Reliability Guided | **-340.38** | **47.5** | **33** | **-7.161** | **<.0001** |
| Free Easy High Reliability UnGuided x Free Easy Low Reliability Guided | **-253.37** | **60.2** | **33** | **-4.206** | **0.0271** |
| Free Easy High Reliability UnGuided x Forced Hard Low Reliability Guided | **-516.81** | **69.5** | **33** | **-7.431** | **<.0001** |
| Free Easy High Reliability UnGuided x Free Hard Low Reliability Guided | **-519.10** | **73.7** | **33** | **-7.039** | **<.0001** |
| Free Easy High Reliability UnGuided x Forced Easy High Reliability Guided | -9.66 | 45.6 | 33 | -0.212 | 1.0000 |
| Free Easy High Reliability UnGuided x Free Easy High Reliability Guided | -31.30 | 52.2 | 33 | -0.599 | 1.0000 |
| Free Easy High Reliability UnGuided x Forced Hard High Reliability Guided | -233.53 | 68.8 | 33 | -3.395 | 0.1710 |
| Free Easy High Reliability UnGuided x Free Hard High Reliability Guided | **-424.94** | **64.7** | **33** | **-6.566** | **<.0001** |
| Forced Hard High Reliability UnGuided x Free Hard High Reliability UnGuided | 76.95 | 62.0 | 33 | 1.242 | 0.9997 |
| Forced Hard High Reliability UnGuided x Forced Easy Medium Reliability Guided | 56.94 | 57.0 | 33 | 0.999 | 1.0000 |
| Forced Hard High Reliability UnGuided x Free Easy Medium Reliability Guided | 149.45 | 63.9 | 33 | 2.337 | 0.7699 |
| Forced Hard High Reliability UnGuided x Forced Hard Medium Reliability Guided | **-158.81** | **37.5** | **33** | **-4.239** | **0.0249** |
| Forced Hard High Reliability UnGuided x Free Hard Medium Reliability Guided | **-342.46** | **72.4** | **33** | **-4.728** | **0.0070** |
| Forced Hard High Reliability UnGuided x Forced Easy Low Reliability Guided | -42.01 | 59.4 | 33 | -0.708 | 1.0000 |
| Forced Hard High Reliability UnGuided x Free Easy Low Reliability Guided | 45.00 | 78.3 | 33 | 0.575 | 1.0000 |
| Forced Hard High Reliability UnGuided x Forced Hard Low Reliability Guided | **-218.44** | **53.3** | **33** | **-4.099** | **0.0353** |
| Forced Hard High Reliability UnGuided x Free Hard Low Reliability Guided | -220.73 | 76.4 | 33 | -2.888 | 0.4146 |
| Forced Hard High Reliability UnGuided x Forced Easy High Reliability Guided | **288.71** | **61.5** | **33** | **4.697** | **0.0076** |
| Forced Hard High Reliability UnGuided x Free Easy High Reliability Guided | **267.07** | **61.6** | **33** | **4.333** | **0.0196** |
| Forced Hard High Reliability UnGuided x Forced Hard High Reliability Guided | 64.84 | 48.6 | 33 | 1.333 | 0.9992 |
| Forced Hard High Reliability UnGuided x Free Hard High Reliability Guided | -126.57 | 67.5 | 33 | -1.874 | 0.9570 |
| Free Hard High Reliability UnGuided x Forced Easy Medium Reliability Guided | -20.01 | 57.5 | 33 | -0.348 | 1.0000 |
| Free Hard High Reliability UnGuided x Free Easy Medium Reliability Guided | 72.50 | 54.0 | 33 | 1.341 | 0.9992 |
| Free Hard High Reliability UnGuided x Forced Hard Medium Reliability Guided | **-235.76** | **59.2** | **33** | **-3.982** | **0.0468** |
| Free Hard High Reliability UnGuided x Free Hard Medium Reliability Guided | -419.42 | 62.4 | 33 | -6.720 | <.0001 |
| Free Hard High Reliability UnGuided x Forced Easy Low Reliability Guided | -118.97 | 61.0 | 33 | -1.950 | 0.9384 |
| Free Hard High Reliability UnGuided x Free Easy Low Reliability Guided | -31.96 | 68.2 | 33 | -0.468 | 1.0000 |
| Free Hard High Reliability UnGuided x Forced Hard Low Reliability Guided | **-295.40** | **62.2** | **33** | **-4.750** | **0.0066** |
| Free Hard High Reliability UnGuided x Free Hard Low Reliability Guided | **-297.68** | **45.1** | **33** | **-6.600** | **<.0001** |
| Free Hard High Reliability UnGuided x Forced Easy High Reliability Guided | 211.75 | 61.4 | 33 | 3.446 | 0.1543 |
| Free Hard High Reliability UnGuided x Free Easy High Reliability Guided | 190.11 | 65.2 | 33 | 2.915 | 0.3988 |
| Free Hard High Reliability UnGuided x Forced Hard High Reliability Guided | -12.12 | 61.4 | 33 | -0.197 | 1.0000 |
| Free Hard High Reliability UnGuided x Free Hard High Reliability Guided | **-203.52** | **53.9** | **33** | **-3.773** | **0.0763** |
| Forced Easy Medium Reliability Guided x Free Easy Medium Reliability Guided | 92.51 | 40.3 | 33 | 2.296 | 0.7936 |
| Forced Easy Medium Reliability Guided x Forced Hard Medium Reliability Guided | **-215.75** | **55.0** | **33** | **-3.926** | **0.0535** |
| Forced Easy Medium Reliability Guided x Free Hard Medium Reliability Guided | **-399.40** | **71.2** | **33** | **-5.613** | **0.0006** |
| Forced Easy Medium Reliability Guided x Forced Easy Low Reliability Guided | -98.95 | 30.5 | 33 | -3.246 | 0.2279 |
| Forced Easy Medium Reliability Guided x Free Easy Low Reliability Guided | -11.94 | 54.4 | 33 | -0.220 | 1.0000 |
| Forced Easy Medium Reliability Guided x Forced Hard Low Reliability Guided | **-275.38** | **49.6** | **33** | **-5.557** | **0.0007** |
| Forced Easy Medium Reliability Guided x Free Hard Low Reliability Guided | **-277.67** | **60.0** | **33** | **-4.627** | **0.0091** |
| Forced Easy Medium Reliability Guided x Forced Easy High Reliability Guided | **231.77** | **30.1** | **33** | **7.700** | **<.0001** |
| Forced Easy Medium Reliability Guided x Free Easy High Reliability Guided | **210.13** | **42.2** | **33** | **4.980** | **0.0035** |
| Forced Easy Medium Reliability Guided x Forced Hard High Reliability Guided | 7.90 | 44.9 | 33 | 0.176 | 1.0000 |
| Forced Easy Medium Reliability Guided x Free Hard High Reliability Guided | -183.51 | 56.2 | 33 | -3.265 | 0.2200 |
| Free Easy Medium Reliability Guided x Forced Hard Medium Reliability Guided | **-308.26** | **58.5** | **33** | **-5.268** | **0.0016** |
| Free Easy Medium Reliability Guided x Free Hard Medium Reliability Guided | **-491.91** | **66.8** | **33** | **-7.368** | **<.0001** |
| Free Easy Medium Reliability Guided x Forced Easy Low Reliability Guided | **-191.46** | **38.6** | **33** | **-4.962** | **0.0037** |
| Free Easy Medium Reliability Guided x Free Easy Low Reliability Guided | -104.46 | 47.1 | 33 | -2.216 | 0.8358 |
| Free Easy Medium Reliability Guided x Forced Hard Low Reliability Guided | **-367.90** | **55.3** | **33** | **-6.658** | **<.0001** |
| Free Easy Medium Reliability Guided x Free Hard Low Reliability Guided | **-370.18** | **54.7** | **33** | **-6.770** | **<.0001** |
| Free Easy Medium Reliability Guided x Forced Easy High Reliability Guided | 139.25 | 41.0 | 33 | 3.398 | 0.1702 |
| Free Easy Medium Reliability Guided x Free Easy High Reliability Guided | 117.61 | 44.2 | 33 | 2.664 | 0.5598 |
| Free Easy Medium Reliability Guided x Forced Hard High Reliability Guided | -84.61 | 52.8 | 33 | -1.604 | 0.9916 |
| Free Easy Medium Reliability Guided x Free Hard High Reliability Guided | **-276.02** | **50.7** | **33** | **-5.449** | **0.0010** |
| Forced Hard Medium Reliability Guided x Free Hard Medium Reliability Guided | -183.65 | 60.5 | 33 | -3.035 | 0.3299 |
| Forced Hard Medium Reliability Guided x Forced Easy Low Reliability Guided | 116.80 | 61.1 | 33 | 1.911 | 0.9485 |
| Forced Hard Medium Reliability Guided x Free Easy Low Reliability Guided | 203.81 | 76.5 | 33 | 2.663 | 0.5602 |
| Forced Hard Medium Reliability Guided x Forced Hard Low Reliability Guided | -59.63 | 53.4 | 33 | -1.116 | 0.9999 |
| Forced Hard Medium Reliability Guided x Free Hard Low Reliability Guided | -61.92 | 64.3 | 33 | -0.962 | 1.0000 |
| Forced Hard Medium Reliability Guided x Forced Easy High Reliability Guided | **447.52** | **64.4** | **33** | **6.945** | **<.0001** |
| Forced Hard Medium Reliability Guided x Free Easy High Reliability Guided | **425.88** | **63.9** | **33** | **6.663** | **<.0001** |
| Forced Hard Medium Reliability Guided x Forced Hard High Reliability Guided | **223.65** | **46.6** | **33** | **4.798** | **0.0058** |
| Forced Hard Medium Reliability Guided x Free Hard High Reliability Guided | 32.24 | 57.6 | 33 | 0.560 | 1.0000 |
| Free Hard Medium Reliability Guided x Forced Easy Low Reliability Guided | **300.45** | **71.6** | **33** | **4.198** | **0.0276** |
| Free Hard Medium Reliability Guided x Free Easy Low Reliability Guided | **387.46** | **82.1** | **33** | **4.718** | **0.0072** |
| Free Hard Medium Reliability Guided x Forced Hard Low Reliability Guided | 124.02 | 80.2 | 33 | 1.546 | 0.9946 |
| Free Hard Medium Reliability Guided x Free Hard Low Reliability Guided | 121.74 | 58.1 | 33 | 2.096 | 0.8894 |
| Free Hard Medium Reliability Guided x Forced Easy High Reliability Guided | **631.17** | **74.4** | **33** | **8.486** | **<.0001** |
| Free Hard Medium Reliability Guided x Free Easy High Reliability Guided | **609.53** | **73.3** | **33** | **8.320** | **<.0001** |
| Free Hard Medium Reliability Guided x Forced Hard High Reliability Guided | **407.30** | **70.9** | **33** | **5.748** | **0.0004** |
| Free Hard Medium Reliability Guided x Free Hard High Reliability Guided | **215.89** | **50.6** | **33** | **4.271** | **0.0230** |
| Forced Easy Low Reliability Guided x Free Easy Low Reliability Guided | 87.01 | 52.8 | 33 | 1.649 | 0.9884 |
| Forced Easy Low Reliability Guided x Forced Hard Low Reliability Guided | **-176.43** | **44.5** | **33** | **-3.964** | **0.0489** |
| Forced Easy Low Reliability Guided x Free Hard Low Reliability Guided | -178.72 | 58.6 | 33 | -3.048 | 0.3230 |
| Forced Easy Low Reliability Guided x Forced Easy High Reliability Guided | **330.72** | **43.5** | **33** | **7.609** | **<.0001** |
| Forced Easy Low Reliability Guided x Free Easy High Reliability Guided | **309.08** | **52.8** | **33** | **5.854** | **0.0003** |
| Forced Easy Low Reliability Guided x Forced Hard High Reliability Guided | 106.85 | 59.3 | 33 | 1.802 | 0.9705 |
| Forced Easy Low Reliability Guided x Free Hard High Reliability Guided | -84.56 | 62.4 | 33 | -1.355 | 0.9990 |
| Free Easy Low Reliability Guided x Forced Hard Low Reliability Guided | -263.44 | 57.0 | 33 | -4.624 | 0.0092 |
| Free Easy Low Reliability Guided x Free Hard Low Reliability Guided | **-265.72** | **62.5** | **33** | **-4.249** | **0.0243** |
| Free Easy Low Reliability Guided x Forced Easy High Reliability Guided | **243.71** | **55.1** | **33** | **4.421** | **0.0157** |
| Free Easy Low Reliability Guided x Free Easy High Reliability Guided | 222.07 | 64.0 | 33 | 3.470 | 0.1469 |
| Free Easy Low Reliability Guided x Forced Hard High Reliability Guided | 19.84 | 67.0 | 33 | 0.296 | 1.0000 |
| Free Easy Low Reliability Guided x Free Hard High Reliability Guided | -171.57 | 69.8 | 33 | -2.459 | 0.6955 |
| Forced Hard Low Reliability Guided x Free Hard Low Reliability Guided | -2.28 | 66.6 | 33 | -0.034 | 1.0000 |
| Forced Hard Low Reliability Guided x Forced Easy High Reliability Guided | **507.15** | **62.5** | **33** | **8.112** | **<.0001** |
| Forced Hard Low Reliability Guided x Free Easy High Reliability Guided | **485.51** | **65.7** | **33** | **7.392** | **<.0001** |
| Forced Hard Low Reliability Guided x Forced Hard High Reliability Guided | **283.28** | **56.7** | **33** | **4.993** | **0.0034** |
| Forced Hard Low Reliability Guided x Free Hard High Reliability Guided | 91.87 | 70.3 | 33 | 1.306 | 0.9994 |
| Free Hard Low Reliability Guided x Forced Easy High Reliability Guided | **509.43** | **68.0** | **33** | **7.493** | **<.0001** |
| Free Hard Low Reliability Guided x Free Easy High Reliability Guided | **487.79** | **71.3** | **33** | **6.846** | **<.0001** |
| Free Hard Low Reliability Guided x Forced Hard High Reliability Guided | **285.57** | **70.7** | **33** | **4.037** | **0.0410** |
| Free Hard Low Reliability Guided x Free Hard High Reliability Guided | 94.16 | 54.0 | 33 | 1.745 | 0.9787 |
| Forced Easy High Reliability Guided x Free Easy High Reliability Guided | -21.64 | 30.9 | 33 | -0.700 | 1.0000 |
| Forced Easy High Reliability Guided x Forced Hard High Reliability Guided | **-223.87** | **47.1** | **33** | **-4.749** | **0.0066** |
| Forced Easy High Reliability Guided x Free Hard High Reliability Guided | **-415.28** | **56.2** | **33** | **-7.388** | **<.0001** |
| Free Easy High Reliability Guided x Forced Hard High Reliability Guided | **-202.23** | **47.6** | **33** | **-4.252** | **0.0241** |
| Free Easy High Reliability Guided x Free Hard High Reliability Guided | **-393.64** | **51.1** | **33** | **-7.698** | **<.0001** |
| Forced Hard High Reliability Guided x Free Hard High Reliability Guided | -191.41 | 51.0 | 33 | -3.754 | 0.0796 |
